# Vernalization regulatory network identifies potential novel functions for genes in the *HvVRN2* locus

**DOI:** 10.1111/nph.71162

**Published:** 2026-04-12

**Authors:** Francesc Montardit‐Tarda, Irene Puyó, Bruno Contreras‐Moreira, Ildikó Karsai, Philippa Borrill, Ana M. Casas, Ernesto Igartua

**Affiliations:** ^1^ Estación Experimental de Aula Dei – Consejo Superior de Investigaciones Científicas (EEAD‐CSIC) Avenida Montañana 1005 50059 Zaragoza España; ^2^ Agricultural Institute Centre for Agricultural Research, Hungarian Research Network Brunszvik u. 2 2462 Martonvásár Hungary; ^3^ Department of Crop Genetics, John Innes Centre Norwich Research Park Norwich NR4 7UH UK

**Keywords:** barley, gene regulatory network, protein modelling, transcriptomic, vernalization, VRN2

## Abstract

- Flowering is controlled by environmental and genetic factors. Vernalization requirement in barley (*Hordeum vulgare* L.) is determined by the allele in *HvVRN1* and the presence of locus *HvVRN2*, which consists of two zinc‐finger and CONSTANS‐like domains (*ZCCT)* genes: *HvVRN2a* and *HvVRN2b*. While the effect of *HvVRN2* presence is well known, its outcome on the transcriptome and other genes outside the flowering/vernalization pathway remains unknown.
- Near‐isogenic lines, with presence/absence of *HvVRN2*, were subjected to vernalization treatments of different lengths, enabling the assessment of *HvVRN2* expression dynamics as well as broader transcriptional responses regulated by VRN2.
- Unknown differences in expression levels of the two *HvVRN2* genes were identified, as well as an effect on tillering. A regulatory network analysis pointed at new candidate genes for flowering regulation related to the vernalization pathway and suggested additional biological roles for *HvVRN2b*. Finally, protein modelling resulted in higher VRN2‐Nuclear Factor‐Y (NF‐Y) stability than other CCT‐NF‐Y complexes.
- This study advances in the understanding of *HvVRN2* in barley adaptation. The *ZCCT* genes within the *HvVRN2* locus should be regarded as distinct genes and may possess broader adaptive functions than previously recognized. Flowering repression by *HvVRN2* may take place at the protein level, potentially inhibiting formation of other CCT/NF‐YB/NF‐YC complexes that activate *HvFT1* transcription.

## Introduction

The transition to flowering is a major physiological event in plants. Its timing has a large agronomic impact on cereal crops of the *Pooideae* family (Carrera *et al*., 2024). This process is highly controlled by environmental conditions and both gene diversity and regulation. Vernalization is the induction of flowering after exposure to a prolonged cold period. This period is necessary in regions with cold winters, in which flowering too early will expose the reproductive tissues to frost risk. During reproductive development, the frost‐sensitive floral structures within the elongating tillers are highly exposed to ambient temperatures. Hence, the need to have a built‐in genetic mechanism to delay this phase until conditions are favourable. Barley and wheat varieties that require vernalization are named winter growth habit genotypes (or ‘winter’ for short).

The vernalization response is elicited by specific allelic combinations at vernalization‐related loci. Three main vernalization‐related genes have been identified in barley: *HvVRN1*, *HvVRN2* and *HvVRN3*. The gene *HvVRN1*, or *HvBM5*, is located on chromosome 5H and encodes for a MADS‐box transcription factor that induces flowering (Trevaskis *et al*., 2003; Yan *et al*., 2003), binding directly to the promoter region of *HvVRN3* (Deng *et al*., 2015). The expression of *HvVRN1* in winter genotypes is activated by enough vernalization time (Trevaskis *et al*., 2006). Two paralogous genes of *HvVRN1*, *HvBM3* and *HvBM8*, are found in most cereals and are considered redundant flowering‐regulating genes (Li *et al*., 2019). *HvVRN2*, located on chromosome 4H, comprises two genes duplicated in tandem, with zinc‐finger and CONSTANS‐like domains (ZCCT) (Yan *et al*., 2004; Dubcovsky *et al*., 2005). A third truncated gene is included in the locus but is not expressed (Trevaskis *et al*., 2006). *HvVRN2* is expressed in leaves during long days, inhibiting *HvVRN3*. After vernalization, *HvVRN1* is activated, which then represses *HvVRN2* in the leaves (Sasani *et al*., 2009), allowing long‐day (LD) induction of *HvVRN3*. The known allelic variation of *HvVRN2* in barley is of presence/absence nature, determining the growth habit of the genotypes: winter (presence) and spring (absence). Moreover, the presence of *VRN2* affects agronomic performance in barley (Karsai *et al*., 2006) and early tiller development in *Triticum aestivum* (Hirsz *et al*., 2026). The ortholog of *HvVRN2* in rice, *Ghd7*, also affects yield components such as grain size and tiller number, besides flowering time (Xue *et al*., 2008; Weng *et al*., 2014). *HvVRN3*, or *HvFT1*, is the orthologue of *Arabidopsis thaliana FLOWERING LOCUS T* (*FT*), promoting the transition to flowering when induced in leaves (Yan *et al*., 2006; Faure *et al*., 2007). *HvFT1* is not expressed in apices (Digel *et al*., 2015). Rather, the protein is transported from the leaves to the apex through the phloem, as evidenced in *Arabidopsis* (Corbesier *et al*., 2007) and rice (Tamaki *et al*., 2007), inducing the reproductive development of the apex due to the florigen signal. Differences in vernalization requirements are mostly modulated by the allelic diversity at these three main vernalization genes, as reviewed in Fernández‐Calleja *et al*. (2021).

Even though not considered as classic vernalization genes, CONSTANS‐like domain containing genes, *HvCO1* and *HvCO2* (Campoli *et al*., 2012), have been proposed to interact with *HvVRN2. HvCO2* is an activator of flowering under LD photoperiod (Mulki & von Korff, 2016). In *Arabidopsis*, CO protein is stable at the end of the day under LD photoperiod, while it is degraded during the morning and night (Brambilla & Fornara, 2017). The CO protein interacts with the dimer formed by Nuclear Factor‐Y (NF‐Y) subunits NF‐YB and NF‐YC, and the resulting complex recognises specifically the CCACA DNA motif, through the CONTANS‐like (CCT) domain (Gnesutta *et al*., 2017). Several CO/NF‐YB/NF‐YC complexes bind to the various CCACA motifs located in the promoter region of *FT*, interacting among them by the two B‐box zinc finger domains of the CO protein, activating *FT* expression (Huang *et al*., 2025). Allelic variation at another photoperiod gene, *HvPRR37* (*PPD*‐*H1*) (Turner *et al*., 2005), has been found responsible for altered expression of *HvVRN2* (Mulki & von Korff, 2016). The genes, *HvCO2*, both *HvVRN2* and *HvPRR37*, present one CCT domain in their protein structure while showing a different number of zinc‐finger domains: two B‐box zinc fingers, one zinc finger and none, respectively. Interactions between these genes and NF‐Y subunits were identified in wheat, using yeast two‐ and three‐hybrid assays. VRN2 protein competes with CO2 for interactions with the same NF‐Y subunits (Li *et al*., 2011). This competition, and the known roles of VRN2 as repressor and CO2 as activator, suggest an antagonistic role of the two types of complexes in progress towards flowering. Li *et al*. (2011) also found that both CO2 and VRN2 could dimerize, as homo‐ and heterodimers, but their effect on *FT1* expression is unknown. PPD1 can dimerize with CO1 and CO2 proteins, but it does not interact with most of the NF‐Y subunits (Shaw *et al*., 2020). The potential role of the CCT protein dimers is not evidenced.

Although transcriptome analysis has long been used in barley (Zhang *et al*., 2004), the advent of affordable RNA sequencing has facilitated the expansion of such studies to encompass multiple tissues and conditions (Cantalapiedra *et al*., 2017; Müller *et al*., 2020). Large‐scale experiments have been conducted to identify transcriptomic differences or landscapes (Thiel *et al*., 2021; Kovacik *et al*., 2024). The availability of transcriptomic data allowed creating expression databases referenced to the barley reference cultivar Morex (Mascher *et al*., 2021), as BarleyExpDB (Li *et al*., 2023), or for the barley reference transcriptome BaRTv.1.0 (Rapazote‐Flores *et al*., 2019), as EoRNA (Milne *et al*., 2021). Therefore, most studies are referenced to the genome of Morex, a spring cultivar lacking the gene models of *HvVRN2*. There is abundant information on the physiological effects of *HvVRN2* on the vernalization mechanism. However, there are still knowledge gaps about gene regulatory networks (GRN) inducing flowering in barley, particularly on the involvement of *HvVRN2*. Genes related to *HvVRN2* could be potential targets for barley breeding aiming at fine‐tuning flowering and improving agronomic performance. We conducted a multifactorial experiment with *HvVRN2* near‐isogenic lines (NILs) and growth conditions chosen to capture the full range of *HvVRN2* expression dynamics, focussing on the transition from vegetative to reproductive stage. The transcriptome of multiple samples was analysed to reveal the GRN involving *HvVRN2*. Protein modelling of CCT genes with NF‐Y subunits was conducted to suggest potential competition of the CCT/NF‐Y complexes and its relevance for flowering regulation.

## Materials and Methods

### Plant material, experimental design and tissue harvest

Two BC_1_‐F1 derived doubled haploid NILs provided by Dr Ben Trevaskis (CSIRO, Australia) were used in this study. The two NILs, CSIRO01 (C01) and CSIRO03 (C03), differed in the presence/absence of the vernalization gene *HvVRN2*, respectively, while sharing the same alleles at *HvVRN1*, *HvPRR37 (PPD‐H1)* and *HvPHYC*. C01 is a true winter genotype (winter allele at *HvVRN1*, presence of *HvVRN2*), whereas C03 is a facultative genotype (winter allele at *HvVRN1*, absence of *HvVRN2*). C01 and C03 are sister doubled haploid lines derived from the backcross of cultivar WI3806 (recurrent) and line GH‐129 (a doubled haploid line from population Galleon × Haruna Nijo), using marker‐assisted selection on chromosomes 2H and 5H. This scheme was performed within a breeding programme aiming at increasing reproductive frost tolerance, which investigated the effect of major flowering time genes. The *HvVRN2 presence* allele came from cultivar WI3806 (Reinheimer, 2010), although no selection for this gene was performed via marker‐assisted selection. Seeds of each genotype were checked with markers for flowering‐related genes and purified with one generation of self‐fertilization. Both genotypes share 97.65% of the genomic background, identified by the 50 K Illumina single nucleotide polymorphism (SNP) chip (Ochagavía *et al*., 2022).

**Fig. 1:**
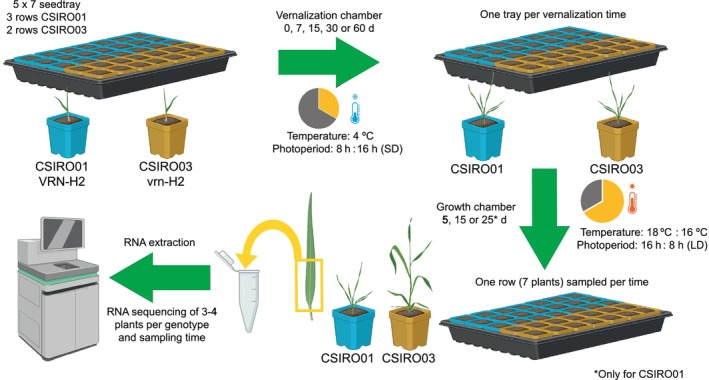
Scheme of the experimental design. Seeds of near‐isogenic lines (NILs) differing in presence/absence of *HvVRN2*, CSIRO01 (C01) with *HvVRN2* and CSIRO03 (C03) without *HvVRN2* were sown in seed trays. After seedling emergence, trays were moved to a vernalization chamber at 4°C and short‐day photoperiod for 7, 15, 30 or 60 d. After the vernalization treatment, plants were grown in standard growing conditions with long‐day photoperiod. A tray without vernalization, considered as 0 d of vernalization, was moved to the growth chamber after seedling emergence. The basal part of the last‐fully expanded leaf from the main tiller of 7 individual plants per NIL was sampled at 5 and 15 d, and at 25 d for C01 due to its slower development. RNA was extracted for all leaf samples, and between 3 and 4 were sequenced per genotype, vernalization treatment and sampling time point, except for C01 at 25 d in growing conditions after 60 d of vernalization with two sequenced samples. Partially created in BioRender (https://BioRender.com/ji42f4i).

*Hordeum vulgare* L. seeds were sown in 5 × 7 seed trays of 200 cm^3^ each cell (115 mm height, 50 × 50 mm at the top and 31 × 31 mm at the bottom), in a soil mix of 47% peat moss, 39% sand and 14% vermiculite, with 0.004% of controlled‐release fertilizer (Plantacote® Plus 4M; 14‐9‐15N‐P‐K plus 2 Mg_2_O and trace elements). After 3–4 d for seedling emergence, the trays were moved to a vernalization chamber at 4 ± 2°C with a short‐day photoperiod of 8 h : 16 h, light : dark, respectively. Plants were vernalized for either 0, 7, 15, 30 or 60 d, and then moved to a growth chamber under LD photoperiod, 16 h : 8 h, 18°C : 16°C, light : dark periods, a relative humidity of 65% and light intensity of 300 μmol m^−2^ s^−1^ photosynthetically active radiation. The seed tray of 0 d of vernalization (no vernalization) was directly placed in the growth chamber after seedling emergence. Each seed tray corresponded to a vernalization time. After 5 or 15 d in the growth chamber, the basal part of the last‐fully expanded leaf from the main tiller, of 7 individual plants, was sampled at 12 h into the light period, and immediately frozen in liquid nitrogen. For genotype C01, with presence of *HvVRN2*, another sample was taken after 25 d. C03 was not sampled at 25 d, as previous experiments indicated that, at this time, the growth stage of this genotype was far more advanced than that of C01 at any vernalization treatment. Each plant was sampled only once, and plants were not removed from the tray. Three of the five tray rows were sown with C01, and each one was completely sampled after 5, 15 or 25 d in the growth chamber. The remaining two rows were sown with C03, and sampled at 5 and 15 d. Although trays (vernalization treatment) were randomized, the rows of the trays could present a nested spatial effect as they were sampled for RNA extraction following the same distribution pattern across trays (from outer to inner rows). Samples were stored at −80°C until RNA extraction. A summary of the experimental design is shown in Fig. 1.

Phenotypic data on apex development, leaf and tiller number for the two genotypes were measured in a separate experiment under two treatments, full or no vernalization, at the phytotron facilities at Martonvásar (Hungary) as partially reported in Ochagavía *et al*. (2022). Four plants per genotype were vernalized at 4°C for 60 d and then moved to the phytotron, in LD photoperiod of 16 h : 8 h, day : night at constant temperature of 18°C. Another four plants per genotype were grown in the phytotron without any vernalization. Following the Zadoks scale (Zadoks *et al*., 1974), the day when plants reached first node appearance, stage Z31, and when awns were visible, stage Z49, was annotated. Leaves on the main tiller and tiller number were counted every 3 to 5 d until stage Z49. At selected dates, main tillers of 2 plants per genotype and treatment were dissected to take pictures of the developing apex, and record the Waddington stage (Waddington *et al*., 1983). Since unvernalized C01 did not reach Z31, tillers were counted until its number was stable in three consecutive measures (which occurred on Day 54). Days to Z31 and Z49, leaf number and phyllochron were already reported, whereas tiller number was not included in Ochagavía *et al*. (2022). Statistical differences between vernalized genotypes at Z31 and Z49 were analysed with a t‐test. Linear models for tiller formation and leaf appearance were fitted per genotype and vernalization treatment. Statistical analyses were computed in the *R* software v.4.4.2 (R Core Team, 2023) and Genstat 22 (VSN International, 2022).

### RNA extraction, sequencing and quality control

RNA was extracted from the leaf samples with the Total RNA Mini Kit for Plants (IBI Scientific, Dubuque, IA, USA), following manufacturer instructions, and quantified using a NanoDrop 2000 (Thermo Fisher Scientific, Waltham, MA, USA). Quality of the RNA extraction was checked in a 1% agarose gel and determined with QUBIT RNA IQ. RNA samples were diluted at 50–100 ng μl^−1^ when necessary and sent to Novogene UK for sequencing. After reception, RNA was quality‐checked with Agilent Bioanalyzer. Libraries were prepared with poly‐A enrichment and quantified with QUBIT and real‐time PCR. Samples were sequenced by Illumina NovaSeq 6000, sequencing of 2 × 150 paired‐end reads for a total of 15‐Gb raw data per sample on average. Raw reads were processed with the fastp software (Chen *et al*., 2018), removing reads with adapters, with *n* ≥ 10% or with a QScore ≤ 5 of over 50% bases of the total bases. All samples were taken in 3–4 biological replicates, with the exception of two replicates for sample C01 at 60 d of vernalization and 25 d LD conditions (Table S1).

### Transcriptome quantification and differentially expressed genes

Clean sequence data was pseudo‐aligned with the kallisto software v.0.46.1 (Bray *et al*., 2016) to BaRT2v.18 reference transcriptome (Coulter *et al*., 2022), of the two‐rowed spring barley cultivar Barke. The reference was chosen for two reasons. First, the Barke transcriptome was built with long reads with a better transcript resolution. Second, C01 and C03 are two‐rowed, genetically closer to Barke than to the common barley reference Morex, which is six‐rowed (Table S2). Since both Barke and Morex lack *HvVRN2*, the transcripts encoded by the two gene models of *HvVRN2* (Horvu_13942_4H01G516500 and Horvu_13942_4H01G516600, identified as *HvVRN2a* and *HvVRN2b*, respectively) were taken from barley pangenome v.1 (https://doi.org/10.5447/ipk/2020/24, Jayakodi *et al*., 2020), specifically from the Spanish winter landrace HOR_13942 (ERS4201453), and added to the reference transcriptome. Transcript‐level estimated counts were normalized into transcripts per million (tpm) (Table S3), and then aggregated to gene‐level with tximport package v.1.34.0 (Soneson *et al*., 2015) in the *R* software v.4.4.2. Uncertainty of the estimated expression was considered with 100 bootstraps.

Principal component analysis (PCA) and identification of differentially expressed genes (DEGs) were carried out with sleuth package v.0.30.1 (Pimentel *et al*., 2017). Genes considered for analysis required at least five estimated counts in 25% of the samples. DEGs were identified with a likelihood ratio test comparing the following two models, aiming to pinpoint DEGs specifically influenced by vernalization:

reduced model=days under longphotoperiod

$$\text{full model}=\begin{array}{l} \text{days under long}\;\text{photoperiod}\\ +\;\text{vernalization} \end{array}$$

The analysis was carried out for the two genotypes separately. A third model was applied to samples of both genotypes at 5 and 15 d under long‐day photoperiod with all vernalization treatments, considering genotype as an additional factor:

$$\text{reduced model}=\begin{array}{l} \text{genotype}+\text{days under long}\;\text{photoperiod} \end{array}$$

$$\text{full model}=\begin{array}{l} \text{genotype}+\text{days under long}\;\text{photoperiod}\\ +\;\text{vernalization} \end{array}$$

Genes were considered as differentially expressed by a multiple test correction adjusted *p*‐val (*q*‐val < 0.01). To check the direction of the vernalization effect on genes, the expression of the genes in samples of C01 at 5 growing days after 0 or 60 d after vernalization were compared with *R* package DESeq2 v.1.46.0 (Love *et al*., 2014), filtering out genes with read counts < 5, and calculating log_2_ Fold Change and the adjusted p‐val.

### Introgression mapping

NILs were genotyped with 50 k Illumina Infinium SNP Array (Bayer *et al*., 2017), referenced to the third version of the Morex reference genome (Mascher *et al*., 2021). Additionally, the clean sequence data were used for fine‐mapping introgression regions. Reads of individual samples were aligned with *HISAT2* (Kim *et al*., 2019) to the Barke v.1 reference genome supplemented with two contigs harbouring two *HvVRN2* gene models from landrace HOR_13942 as explained earlier. Variant calling was performed with bcftools v.1.17–53 (Li, 2011), filtering biallelic variants with a QUAL > 50 and identified by at least 10 reads. Heterozygous calls were considered as missing values. SNPs were considered robust if present in more than 50% of the samples per genotype, and with the same allele. Graphical genotypes representing the introgressions were plotted with chromomap package v.4.1.1 (Anand & Rodriguez Lopez, 2022).

### Identification of co‐expressed genes

Clustering in modules of co‐expression was performed at the gene‐level by using the uwgcna package v.1.73 (Langfelder & Horvath, 2008), which uses a correlation network approach. Genes were selected if they presented 10 counts in at least nine samples. Gene expression in counts was variance‐stabilizing transformed with deseq2 package v.1.46.0 using the parameter blind = TRUE (Love *et al*., 2014). Gene and sample outliers were checked, and filtered out if necessary. A soft‐power threshold was calculated as the first power to exceed a scale‐free topology fit index of 0.9 (β = 4), considering a signed hybrid correlation network and biweight midcorrelations. Parameters for network construction were selected by comparing different combinations, considering the total number of identified modules and flowering‐related genes membership. The topological overlap matrix (TOM) was created with an unsigned TOMtype approach. The minimum number of genes per module was 40 (minSize = 40). Dendrogram was cut at 0.97 for module detection (detectCutHeight = 0.97), but module splitting was set to mid‐sensitive (deepSplit = 2) and module merging was allowed (mergeCutHeight = 0.15). Co‐expression module membership was calculated for all genes. Module eigengene (ME) was calculated, corresponding as the first principal component per module and considered as a representative of its gene expression profile (Langfelder & Horvath, 2008). MEs of all modules were correlated to the factors of the experimental design.

### Gene set enrichment analysis

Co‐expression modules gene sets were enriched by Gene Ontology (GO) terms with clusterprofiler package v.4.16.6 (Wu *et al*., 2021), using the annotations of BaRTv2 complemented with the GO terms of both *HvVRN2* gene models from the HOR_13942 genome annotation.

### Transcription factor annotation

For further analysis, identification of transcription factor (TF) gene models of BaRTv2, including both *HvVRN2* gene models from HOR_13942, was required. Protein sequences of all transcripts were analyzed with the *iTAK* Web server v.18.12 (Zheng *et al*., 2016). Annotation at gene‐level considered the longest transcript.

### Gene regulatory network construction

A gene‐regulatory network was constructed with the expression data, filtered as previously described for co‐expression clustering and a list of transcription factors using a random forest approach, with genie3 package v.1.28.0 (Huynh‐Thu *et al*., 2010). Relationships between genes were filtered with an edge weight ≥ 0.01. Additionally, the network was filtered by known flowering‐related genes (Table S4), to focus on the pathways related to flowering and vernalization, and for interactions between transcription factors Network was constructed with ggally package v.2.2.1 (Schloerke *et al*., 2024) using the Fruchterman–Reingold algorithm, and then manually modified with cytoscape v.3.10.3 tool (Shannon *et al*., 2003).

### Pangenome homology of expressed genes

Collinearity‐based gene homology between the reference transcriptome (BaRTv2) and barley pangenome v.1 was identified with *GET_PANGENES* (Contreras‐Moreira *et al*., 2023) and saved as file pangene_matrix_genes.tr.tab (v.04102024), available at https://github.com/eead‐csic‐compbio/barley_pangenes. The resulting pangene set was also used to plot the genomic context of gene *HvSNF2*, linked to *HvVRN2*, with script check_evidence.pl from *GET_PANGENES*.

### Modelization of protein complex structures

Protein complexes of NF‐Y domains with other proteins carrying a CCT domain (HvCO1, HvCO2, HvVRN2 and HvPRR37) were modelled with Alphafold 3 Web server (Abramson *et al*., 2024). As a proof of concept, HvVRN2a was modelled with other binding partners of Protein Data Bank entries 7C9O and 7CVO, produced for rice (Shen *et al*., 2020) and for *Arabidopsis thaliana* (Lv *et al*., 2021), obtaining high‐scoring complexes. The amino acid sequences encoded by NF‐YB and NF‐YC genes in barley (Panahi *et al*., 2019), enriched with the annotated high‐confidence genes as ‘Nuclear Factor Y’ in the MorexV3 genome (Table S5), were clustered to avoid redundancy with *CD‐HIT* v.4.8.1 (Li & Godzik, 2006), with a sequence identity cut‐off of 0.85. A NF‐Y protein sequence was selected per cluster (12 for NF‐YB and 9 NF‐YC), considered as representative. Multiple sequence alignments (MSA) of all representative NF‐Y against the NF‐YB11 or NF‐YC2 proteins of the *Oryza sativa* Japonica group 7C9O complex were performed with Clustal omega tool v.1.2.4 (Sievers & Higgins, 2014). Sequences were trimmed at the start and end according to the MSA, with 7C9O sequences as reference, to reduce noise added by sequences outside the macrocomplex. Models were created for all combinations of the complexes of NF‐YB and NF‐YC subunits with HvCO1, HvCO2, HvVRN2a or HvPRR37, and an oligonucleotide of length 31 with the CCACA motif extracted from the promoter region of *HvVRN2a* of HOR_13942 (Table S5). The stability of the modelled complexes was calculated as the sum of the values of interface predicted template modeling (iPTM) and predicted template modeling (PTM) metrics, as proposed in Homma *et al*. (2023).

The same procedure was applied to wheat. We focussed on the NF‐Y proteins having complete sequences (3 per NF‐YB and NF‐YC), and evidence of interaction with CCT proteins, reported by Li *et al*. (2011). Before modelling, wheat NF‐YB and NF‐YC proteins were BLASTed to all trimmed sequences of barley NF‐YB or NF‐YC, respectively. Wheat NF‐Y were trimmed based on the alignment to their most similar barley NF‐Y. Combinations of NF‐YB and NF‐YC subunits were tested with VRN2 from *Triticum monococcum* (ZCCT1, Li *et al*., 2011), PPD‐D1, CO‐D1 and CO‐B2 from *T. aestivum*, and a oligonucleotide of length 30 with CCACA motif extracted from the promoter region of *T. aestivum* FT1 (Table S5).

The position of the CCACA motif across the promoter region (−1000 bp, 0 bp) of the genes coding CCT domain proteins were scanned with *matrix‐scan‐quick* of the RSAT software (Santana‐Garcia *et al*., 2022). Promoter sequences were extracted, using the coordinates available at Table S5 from the genome of HOR_13942 (ERS4201453) from the pangenome v.1 (Jayakodi *et al*., 2020).

## Results

### Gene expression changes are mediated by *HvVRN2* and vernalization

To investigate how vernalization affects gene expression through *HvVRN2*, two barley NILS, with *HvVRN2* (C01) or without *HvVRN2* (C03), were exposed to vernalising temperatures for 0, 7, 15, 30 or 60 d before transferring to standard growth conditions for 5, 15 or 25 d (Fig. 1). We hypothesised that these varying conditions would generate quantitative effects on vernalization responses, which could be detected through gene expression changes. Gene expression of the basal part of the youngest fully expanded leaf from the main tiller was measured in each combination of vernalization and growing time per genotype, sequencing mRNA.

**Fig. 2:**
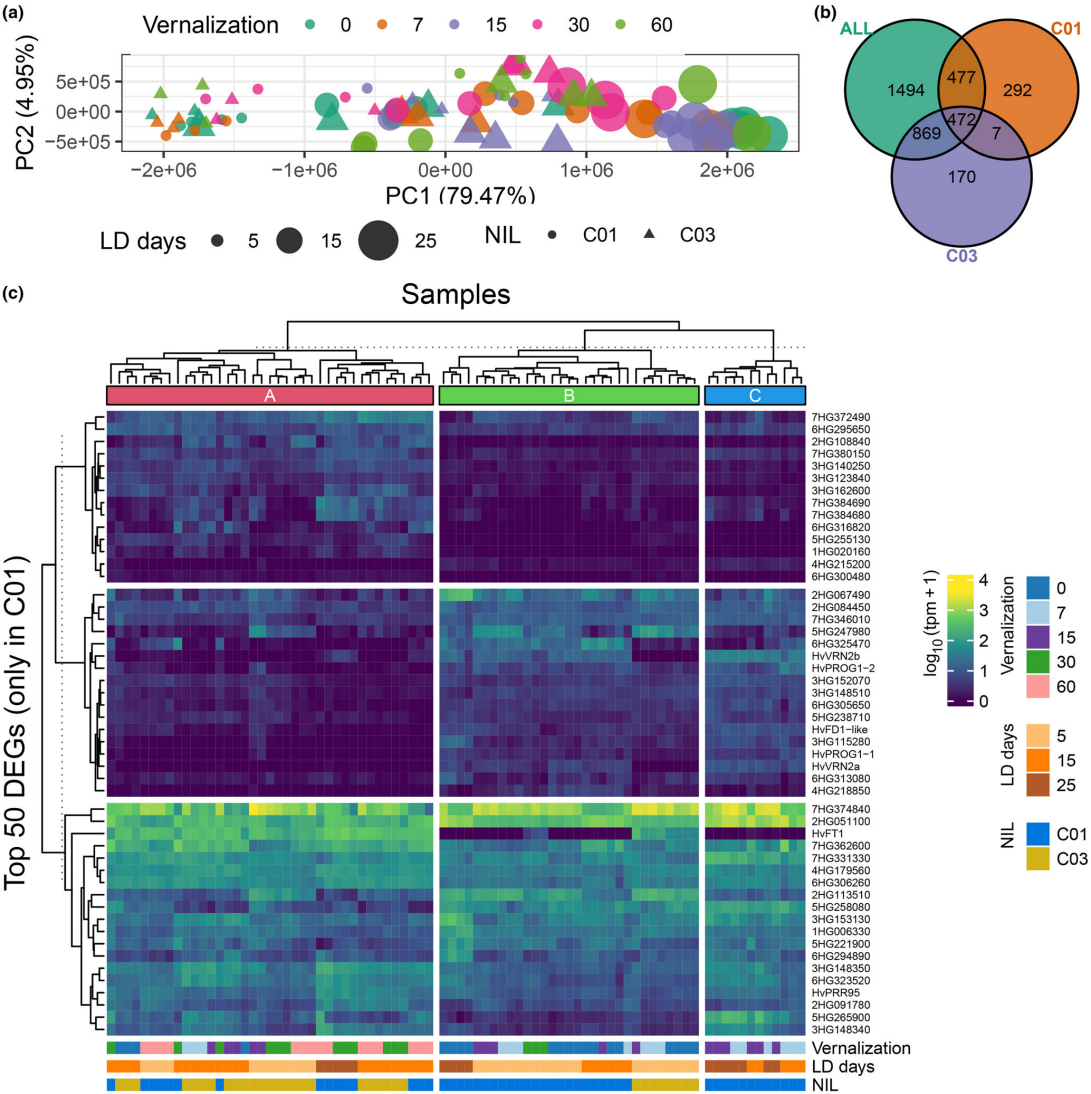
Transcriptome‐level differences between samples and differentially expressed genes (DEGs) by vernalization and presence of *HvVRN2*. (a) Principal component analysis of all samples. *Colour* indicates vernalization time, *size* days in growing conditions at long‐day (LD) photoperiod, and *shape* the near‐isogenic lines C01, genotype with presence of *HvVRN2*, as circles and C03, without *HvVRN2*, as triangles. (b) Venn Diagram of DEGs detected between the three models focussing on vernalization. Green for the ALL model (top‐left), including both genotypes and all vernalization times at 5 and 15 d in LD growing conditions, orange for C01 model (top‐right) and purple for C03 model (bottom), for all available samples of each genotype separately. (c) Heatmap of the logarithmic expression across all samples of the top 50 DEGs by vernalization and presence of *HvVRN2*, identified only in the C01 model or in the intersection between the ALL and C01 models. Samples, in the x‐axis, clustered in three major groups. Genes are identified by either their gene name or their BaRTv2 gene model ID, trimming ‘BaRT2v18chr’ to shorten its length, that is 3HG148340 as BaRT2v18chr3HG148340.

Although the focus of the experiment is vernalization, a PCA revealed that plant age under a LD photoperiod explained more variance than vernalization for both genotypes (Fig. 2a). At the time of sampling, genotypes were in different developmental stages across treatments (Fig. S1). DEGs affected by vernalization were identified combining the samples for both genotypes at 5 and 15 d of growth for all vernalization treatments (model ALL; Fig. 2b), and also for each genotype separately (models C01, CO3). For C01 genotype, samples taken at 25 d of growth were also included. DEGs related to LD growing conditions, and genotype for the combined analyses, were masked using a likelihood ratio test between two fitted models, one with only the LD treatment and another combining the LD treatment with vernalization (Table S3). The total number of DEGs per model was 3312, 1248 and 1518 for the combined model (ALL), C01‐ or C03‐specific models, respectively. The intersection of the sets of DEGs between the three models marked which genes were affected by vernalization alone or by the effect of both *HvVRN2* presence and vernalization. A total of 769 DEGs were identified in C01, and not in C03, which could be the result of the presence and expression of *HvVRN2* (Fig. 2b). The gene with the lowest *q*‐value due to the vernalization factor was *HvFT1* (Table 1), the main integrator of the vernalization and photoperiod pathways. The two *HvVRN2* genes were in third and seventh position, sorting the DEGs by increasing *q*‐value. Out of all DEGs, only 4.8% were in introgressed regions (Table S3; Fig. S2), which is consistent with the identity of the two genotypes of 95.7%.

**Table 1:** Differentially expressed genes (DEGs) influenced by vernalization, selected from DEGs identified in the C01 model, either known previously or newly identified in this study.

BaRT2v ID	Gene ID	Annotation Morex	ALL	C01	C03	WGCNA
BaRT2v18chr7HG338110	*HvFT1*	Flowering locus T	**	**	*	M20
Horvu_13942_4H01G516500	*HvVRN2a*		**	**	–	M0
BaRT2v18chr4HG216300	*HvPROG1‐1*	Zinc finger protein	**	**	–	M18
Horvu_13942_4H01G516600	*HvVRN2b*		*	**	–	M29
BaRT2v18chr6HG305650	*HvbHLH*	Basic helix–loop–helix TF	**	**	–	M27
BaRT2v18chr5HG258900	*HvPRR95*	Pseudo response regulator	**	**	–	M20
BaRT2v18chr4HG216310	*HvPROG1‐2*	Zinc finger protein	**	**	–	M27
BaRT2v18chr3HG115280		Zinc finger family protein	**	**	*	M18
BaRT2v18chr5HG258080	*HvCBF4A*	CRT‐binding factor	**	**	*	M18
BaRT2v18chr4HG215200	*HvTEM1‐like*	AP2/B3 Transcription factor	**	**	*	M18
BaRT2v18chr5HG259930	*HvFD1‐like*	Transcription factor	**	**	–	M18
BaRT2v18chr4HG206880		B3 domain‐containing protein	**	**	–	M20
BaRT2v18chr1HG013580	*HvARF*	Auxin‐responsive protein	–	**	–	M29
BaRT2v18chr4HG219040		GATA transcription factor	**	**	–	M0
BaRT2v18chr5HG265940	*HvVRN1*	MADS box transcription factor	**	**	**	M20
BaRT2v18chr3HG128710	*HvFT2*	Flowering locus T	**	**	**	M20
BaRT2v18chr2HG079040	*HvBM8*	MADS‐box transcription factor	**	**	**	M20
BaRT2v18chr6HG320060	*HvABF4*	BZIP transcription factor	**	**	**	M18
BaRT2v18chr2HG066580	*HvBM3*	MADS box transcription factor	**	**	**	M20
BaRT2v18chr5HG258090	*HvCBF2A*	CRT‐binding factor	**	**	**	M18
BaRT2v18chr2HG056170	*HvPRR37*	Pseudo response regulator	**	**	**	M20
BaRT2v18chr5HG258070	*HvCBF*	CRT‐binding factor	**	**	**	M27
BaRT2v18chr7HG343770	*HvVRT2*	MADS transcription factor	**	**	**	M18

Columns ALL, C01 and C03 indicate the significance of each gene in each model of DEG analysis (**, *q* value < 0.01, *, *q* value < 0.05, −, *q* value ≥ 0.05). The right most column indicates the WGCNA module. Full data are presented in Supporting Information Table S3.

The intersection of the three models (ALL, C01, C03) divided the set of 769 genes into two subsets, one detected already in the ALL model, run with 5 and 15 d samples (477 genes), which can be named *early* response, and another one only detected once the 25‐d samples were taken into account (292 genes), or *delayed* response. The genes most closely related to the action of *HvVRN2* should be in the subset of *early* response. Remarkably, *HvVRN2a* falls in the early subset, whereas *HvVRN2b* is located in the delayed subset. In addition, 479 DEGs shared by both genotypes were equally differentially expressed by the vernalization factor (Tables 1, S3), but *HvVRN2* was likely not involved. A clustering of all samples of both genotypes, according to the top 50 DEGs due to vernalization only identified in C01 samples (Table S3), resulted in three major groups (Fig. 2c). These three clusters correspond to three developmental moments: plants still in vegetative stage, either young (5 d) or C01 without vernalization (B, green); plants in reproductive stage (A, red), either C03 or C01 after at least 30 d of vernalization; and C01 samples partially vernalized (7 and 15 d), collected after 15 and 25 d. In these samples, the transition to reproductive phase was still blocked due to the expression of both *HvVRN2* genes, but their independent clustering indicates that these samples had undergone gene expression changes beyond those observed in plants in vegetative state (cluster B).

### *HvVRN2* genes are repressed by enough vernalization but activated by partial vernalization

**Fig. 3:**
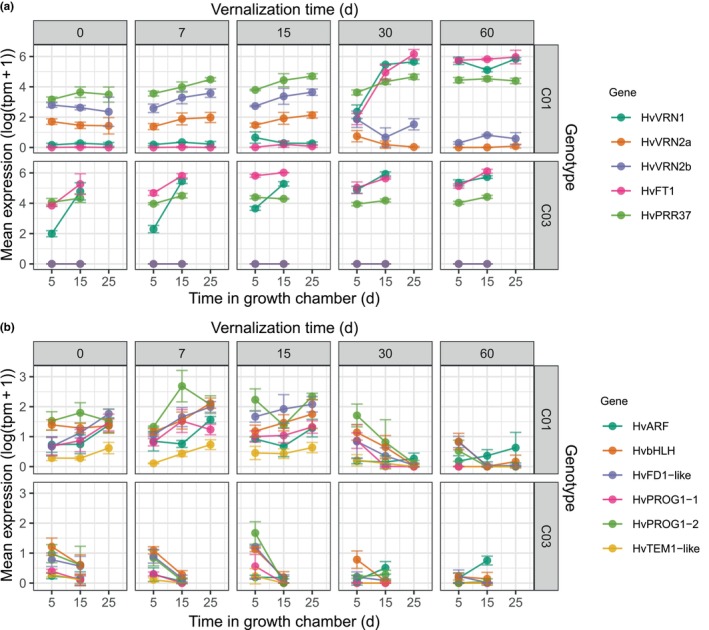
Expression of relevant flowering‐related genes in both genotypes. Values are the mean expression of the logarithm of transcripts per million + 1, considering all samples of each timepoint (*n* = 3–4, except C01 at 25 d after 60 d of vernalization with *n* = 2). Error bars indicate SD. Horizontal panels differentiate the near‐isogenic lines, C01 (upper) with presence of *HvVRN2* and C03 (lower) without *HvVRN2*. Each vertical panel is a vernalization time treatment of 0, 7, 15, 30 or 60 d (left to right). Vernalization panels are divided by each of the sampling points at long‐day photoperiod in the growth chamber, at 5 and 15 d. C01 plants were also sampled at 25 d. (a) *HvVRN* genes and main flowering time genes, affected by vernalization; (b) selected genes differentially expressed, affected by vernalization, identified in the gene regulatory network.

Overall, the expression of the two *HvVRN2* genes followed expectations, according to the standing hypotheses that they are induced by long days and repressed by full vernalization. However, two striking differences were found. Both genes maintained a rather high expression in C01, up to the vernalization treatment of 15 d. With longer vernalization, they were repressed more or less proportionally to the increased expression of their repressor, *HvVRN1*. *HvVRN2b* had much higher expression than *HvVRN2a*. Besides, *HvVRN2a* was completely repressed at some time points, whereas *HvVRN2b* showed some expression across all experimental conditions, even well after *HvVRN1* was fully induced and *HvFT1* was already activated (Fig. 3a).

Additionally, we observed a certain induction of the two *HvVRN2* genes due to partial vernalization. According to the standing hypothesis, their expression is induced by long days. However, when plants were partially vernalized (7, 15 d of cold treatment), both *HvVRN2a* and *HvVRN2b* increased steadily in expression, in plants that still did not reach jointing stage (Z31). Expression of both *HvVRN2* genes was already induced at the 5‐d sampling point, when vernalization was low or absent (0, 7 and 15 d of cold treatment). However, it increased significantly over time only when some vernalization was applied (Fig. S3; Table S6). Vernalization treatments of 7 and 15 d provided not enough vernalization to induce *HvVRN1*, but enough to trigger vernalization‐dependent mechanisms that induced *HvVRN2* expression. This increase in expression did not occur in plants that were not exposed to cold conditions (0 d vernalization). On the other hand, *HvVRN2* expression was kept at lower levels after longer vernalization periods (30, 60 d), most likely due to the induction of *HvVRN1* expression. This induction was evident after 30 d of vernalization. At this time point, the regression slope of *HvVRN1* expression over time was significantly different from 0 and from the flat responses of all other vernalization treatments (Fig. S3; Table S6). Taken together, the dynamics of response of the three genes over time as a function of vernalization indicate an induction of *HvVRN2* genes before enough vernalization triggers *HvVRN1* expression, and their repression after *HvVRN1* is expressed. These facts suggest the presence of additional genes related to partial vernalization influence on *HvVRN2* expression.

### Co‐expression clustering identified vernalization‐related gene modules

Co‐expressed genes follow similar patterns of activation/repression as a response to environmental conditions or are regulated by shared TF. Genes were clustered in modules by their co‐expression (Table S3) with weighted correlation network analysis (WGCNA), identifying 30 modules. The module size varied between 61 and 2801 genes and included 50% of the total expressed genes. An extra module, M0, grouped 7558 genes with not enough co‐expression with others (Table 2).

**Table 2:** Co‐expression modules identified by WGCNA, detailing total number of genes, correlation coefficients with the experimental factors (near‐isogenic lines (NIL) or genotype, vernalization and long‐day (LD) days), and number of differentially expressed genes (DEGs) found with two different models used to identify DEGs (ALL, C01).

Module	No. of genes	NIL	Vernalization	LD days	ALL	C01	ALL + C01
M0	7558	−0.29	−0.52	0.37	409	212	77
M1	2801	−0.36	0.13	0.82	213	62	25
M2	2778	−0.19	0.25	0.76	297	40	14
M3	2777	0.42	0.06	−0.82	441	48	21
M4	1170	0.40	0.31	−0.49	97	20	5
M5	1159	−0.28	−0.19	0.76	154	25	8
M6	898	0.19	−0.19	−0.85	220	75	18
M7	793	−0.10	−0.04	0.17	11	2	1
M8	626	−0.10	−0.32	0.35	266	25	5
M9	479	0.30	−0.11	−0.67	9	4	0
M10	451	0.19	−0.36	−0.66	104	26	12
M11	416	0.08	−0.39	−0.27	52	29	13
M12	387	−0.03	−0.63	−0.27	124	107	49
M13	373	−0.21	0.14	0.59	6	1	1
M14	368	0.27	−0.14	−0.44	15	11	1
M15	362	0.24	0.52	−0.48	67	26	7
M16	350	0.10	−0.48	−0.47	85	84	37
M17	345	−0.34	−0.24	0.56	21	1	0
M18	342	−0.37	−0.71	−0.02	117	100	40
M19	342	0.13	−0.52	−0.70	136	77	30
M20	315	0.33	0.75	0.17	128	121	51
M21	302	−0.04	−0.07	0.52	68	3	0
M22	295	−0.27	0.22	0.48	18	21	7
M23	276	0.24	0.42	0.06	28	3	0
M24	258	0.14	−0.08	−0.25	3	1	0
M25	245	0.05	−0.37	−0.20	80	21	10
M26	204	0.23	−0.27	−0.60	40	32	21
M27	167	−0.17	−0.66	−0.47	79	65	22
M28	111	0.99	0.04	−0.30	6	2	2
M29	86	−0.96	−0.04	0.47	2	4	0
M30	61	0.08	0.42	−0.01	16	0	0

The ALL + C01 column indicates the number of DEGs shared between ALL and C01 models, but not identified in the C03 model.

*HvVRN2b* was included in module M29 with another 85 genes, while *HvVRN2a* was in module M0, probably due to its low expression overall. Interestingly, the closest gene to the *HvVRN2* loci on chromosome 4H, the *SNF2 Helicase* (BaRT2v18chr4HG219190), was co‐expressed with *HvVRN2b*. The assignment of known flowering time genes to modules hints at their possible position in plant development. *HvVRN1* was located within module M20, same as other flowering‐related MADS‐box genes *HvBM3* and *HvBM8*. Module M20 contained 315 genes, including several *FT* genes (*HvFT1*, *HvFT2* and *HvFT4*), FD‐like genes (*HvFD‐like 15*), CONSTANS‐like genes (*HvCO2*) and genes related to the circadian clock (*HvPRR95* and *HvPRR37*). Another remarkable module was M18, which included genes such as C‐repeat binding factor (CBFs) (stress response genes), *HvVRT2* (MADS‐box influencing development). Interestingly, DEG BaRT2v18chr4HG216300, also in M18, is one of the orthologues of rice gene *OsPROG1* that controls plant architecture (Jin *et al*., 2008; Tan *et al*., 2008); BaRT2v18chr4HG215200, an AP2/B3 TF; and BaRT2v18chr5HG259930, an orthologue of rice *OsBZIP77 (OsFD1)* related to flowering control (Tsuji *et al*., 2013). Module M27 contained genes such as a basic helix–loop–helix (bHLH) TF, CBF, and a zinc finger protein, BaRT2v18chr4HG216310, another orthologue of rice *OsPROG1*. The patterns of expression of some of these genes reveal their close relation to vernalization (Fig. 3b).

**Fig. 4:**
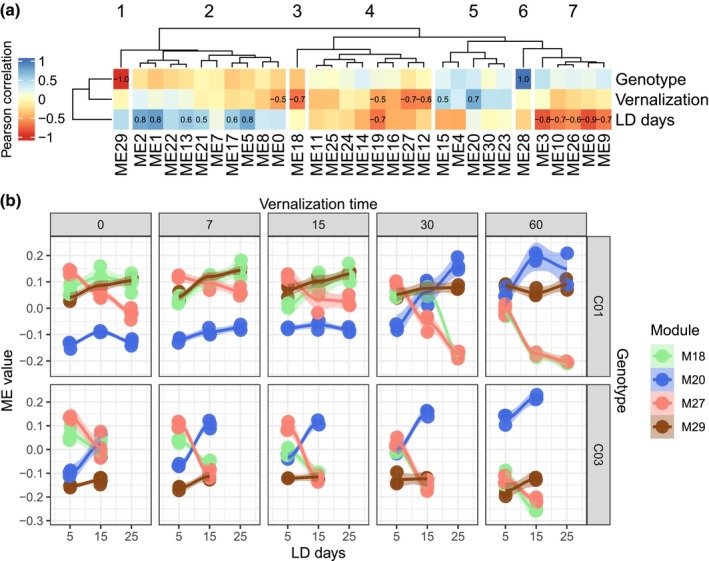
Co‐expression modules most related to vernalization and presence of *HvVRN2* locus. (a) Heatmap of the correlation between co‐expression module eigengenes, considered as representative of the gene expression profile, with the factors used in the experimental design, clustering them in seven groups by dendrogram height. Cell values indicate the correlations above an absolute value of 0.5. The near‐isogenic lines, genotype factor, were converted to a binary value as 0 for C01, with presence of *HvVRN2*, and 1 for C03, without *HvVRN2*. (b) Module eigengenes values related to vernalization (M18, M20 and M27) and presence of *HvVRN2* (M29) throughout all variables of the experimental design: genotype, vernalization time and days of growth under long‐day photoperiod. A smooth line was fitted to visualize differences between samples of each treatment.

A correlation between modules and the three experimental design factors (genotype or presence/absence of *HvVRN2*, time of vernalization and growing days under LD) suggested the factors triggering the expression changes in the modules and, particularly, which modules were mostly related to vernalization and presence of *HvVRN2* (Table 2; Fig. 4a). Modules 18, 20 and 27 presented the highest changes in expression due to vernalization. The first two also showed a negligible effect of plant age (days in LD), and a moderate weight of genotype. These two modules seem to represent the most immediate response to vernalization, in opposite directions: genes of module 18 reducing their expression, whereas those in 20 increase it, in response to increasing vernalization. This is also reflected in the opposite directions followed by M18 and M20 eigengene values as vernalization time increased (Fig. 4b). Clustering the correlation of the modules by the experimental factors identified seven branches in the dendrogram. Branch 4 (Fig. 4a) groups modules with a sizable negative effect of vernalization on gene expression, with a negligible genotypic effect, including Module 27. Modules 18 and 27 sat in different branches, due to the important effect of LD time on expression of genes in Module 27. Branches 2 and 7 (Fig. 4a), on the other hand, presented a low‐to‐moderate relation with vernalization (with the exception of M0), and a stronger relationship with time in LD, in opposite directions (increased expression with time for Branch 2, decreased for Branch 7). M28 and M29 were highly correlated with the genotype background or the absence/presence of *HvVRN2* locus, respectively, but neither bore any relationship to vernalization.

The number of DEGs identified in each module, presented in Table 2, indicates the number of genes involved in response to vernalization possibly driven by the presence of *HvVRN2* in column ALL+C01. The highest proportion of DEGs is in modules M12, M16, M18, M20 and M27. These genes can be easily identified by filtering in Table S3.

GO enrichment of the modules provided some insight into the functions prevalent for each gene cluster (Table S7). The GO enrichment of M20 showed terms related to shoot system development and DNA‐binding transcription factor activity. *HvVRN1* and *HvFT1* were part of this co‐expression module, as were other flowering‐related genes. Even though not highly correlated with vernalization, M23 was clustered alongside M20 and its enrichment resulted in terms related to chloroplasts and, more importantly, to ovary development. M18 was preferentially related to hormonal response.

### The flowering/vernalization gene regulatory network reveals differences in the interactions of *HvVRN2* genes

TFs are major regulators of the floral transition. To identify downstream target genes, we identified a total of 2466 TF from the BaRT2v18 catalogue (Table S8). After filtering, 1806 were part of the expression data set and were used for GRN construction. A GRN indicates the probability of a target gene being regulated by a TF. The GRN identified the interactions between several TF and their target genes, including well‐known regulations (Table S9).

**Fig. 5:**
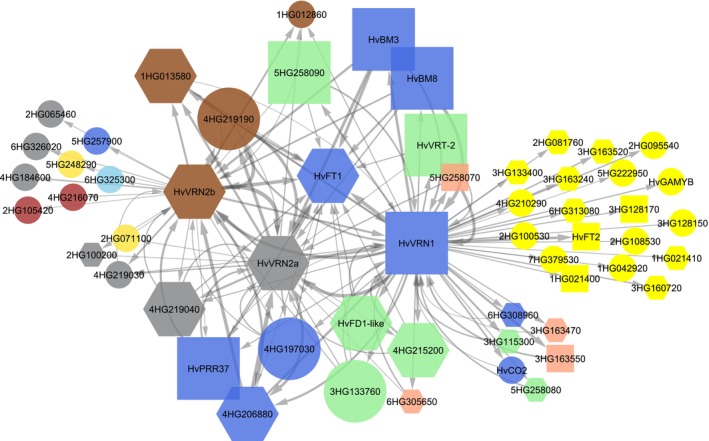
Gene regulatory network (GRN) predicted for the three main vernalization genes *HvVRN1*, *HvVRN2* and *HvFT1* (*HvVRN3*) is similar to the standing hypothesis. The GRN shows only links between transcription factors and those three main genes. Larger node size indicates genes directly connected to *HvFT1*. Node shape determines whether the gene was identified as differentially expressed due to vernalization (*q*‐value < 0.01) in both near‐isogenic lines (square), only in C01 genotype, with presence of *HvVRN2* (hexagon), or was not differentially expressed (circle). Node colour is assigned by co‐expression module: blue for M20, green for M18, brown for M29, salmon for M27, lightblue for M28, darkblue for M15, yellow for M4, red for M3, and orange for M19. Grey colour corresponds to M0, which includes nonclustered genes. Arrow width is proportional to the weight computed by GENIE3. Genes are labelled by their gene name or their gene ID model from BaRT2v18, trimming ‘BaRT2v18chr’ to shorten its length, that is 4HG219190 as BaRT2v18chr4HG219190.

As this study focusses on vernalization, GRN was filtered to include all genic interactions of *HvVRN1*, both *HvVRN2* genes, and *HvFT1* with other TF and FT genes (Figs 5, S4; Table S10). Twelve TF were connected by both *HvVRN1* and at least one of the *HvVRN2*, and also to *HvFT1*. The resulting network displays the genes that are most likely involved in the vernalization and flowering pathways. These genes were mostly from three co‐expression modules: M18, M20 and M29. This is reinforced by the fact that 24 of the 50 most significant DEGs in C01 (Fig. 2c) belonged to M18 and M20, indicating the strongest responses were preferentially grouped in these modules. Some already known vernalization‐ and flowering‐related genes were identified in the GRN as highly related to the vernalization genes, such as *HvBM3* and *HvBM8*, paralogues of *HvVRN1* (Ejaz & von Korff, 2017; Li *et al*., 2019), the circadian clock gene *HvPRR37* or *PPD‐H1* (Turner *et al*., 2005), three CBFs, associated with freezing tolerance (Stockinger *et al*., 2007; Dhillon *et al*., 2010) or the MADS‐box *HvVRT2* (Kane *et al*., 2005; Trevaskis *et al*., 2007).

The inclusion of these genes indicates that the experiment and methodology of analysis followed are able to identify well‐known relationships. Moreover, some new TFs appearing in the network were also identified as differentially expressed only in C01, such as an AP2/B3 (BaRT2v18chr4HG215200), a bHLH (BaRT2v18chr6HG305650), a BZIP77‐like (BaRT2v18chr5HG259930), a B3 domain‐containing protein (BaRT2v18chr4HG206880), an Auxin‐responsive protein (BaRT2v18chr1HG013580) or a GATA TF (BaRT2v18chr4HG219040). The connections between these genes, their co‐expression modules and which experimental factors correlate best with the modules allowed us to propose a temporal sequence, in good correspondence with the underlying hypotheses linking the functions of the well‐known flowering genes found in them (Fig. S5): genes in modules M18 and M29 are induced after exposure to low temperature, possibly with hormone involvement, and expressed when plants are not fully vernalized, while M20 genes are activated when plants do not require vernalization or are fully vernalized, with *HvFT1* as the main example. M18 and M29 regulate the transition of flowering, most likely as repressors. M20 includes inducers of the transition to flowering. When there is enough vernalization, M20 genes are activated, repressing M29 function and the transition to reproductive phase will be effective.

We have found genes apparently coregulated with *HvVRN2b* (M29), and others acting in close association. One of them, *SNF2* (*helicase*, BaRT2v18chr4HG219190), sits next to the *HvVRN2 locus* on chromosome 4H (Fig. S6). However, the alleles of the two NIL genotypes at this gene are different; the CSIRO01 *HvSNF2* allele was likely introgressed during the introduction of *HvVRN2* by linkage drag. The other two genes in the network in the M29 module, an Auxin‐responsive protein (BaRT2v18chr1HG013580) and a Histone‐lysine methyltransferase (BaRT2v18chr1HG012860), are located on chromosome 1H, which is free of off‐target introgressions (Table S3).

The network revealed interactions between *HvVRN1* and other genes, differentially expressed by vernalization, affecting *HvVRN2a* expression, but not *HvVRN2b*. This refers to TFs *AP2/B3*, *HvVRT2*, *HvFD1‐like*, *CBF* and *bHLH*. In addition, *HvVRN2b* affects the expression of other unrelated genes. It is worth mentioning that the largest number of interactions detected was for the MADS‐box gene *HvVRN1*.

### Modelling of CCT/NF‐Y complexes predicted higher stability of VRN2/NF *‐*Y complexes

Genes with CONSTANS‐like (CCT) protein domain are heavily implicated in flowering regulation, integrating the photoperiod (*HvCO1*/*HvCO2* and *HvPRR37*) and vernalization (*HvVRN2*) pathways to control the transition to the reproductive phase. Protein–protein interactions between the CCT genes have been identified *in vitro* and *in vivo* in other species, as well as with NF‐Y subunits, required to trigger the expression of the florigen signal from *FT*. Still, the regulation at the protein level had not been studied in barley. We conducted an *ex situ* approach through protein modelling prediction to check whether the protein–protein interactions were plausible. We investigated the three‐dimensional structure of the complexes with CCT domain proteins, using AlphaFold3. Based on the models constructed for rice Hd1 and Arabidopsis CO proteins (Shen *et al*., 2020; Lv *et al*., 2021), the DNA‐binding domain was the CCT domain rather than the zinc‐finger domains of *HvCO1*/*HvCO2* or both *HvVRN2*.

**Fig. 6:**
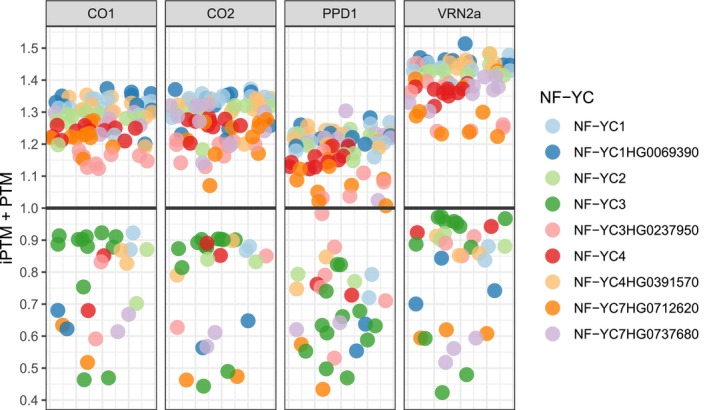
Differences in stability of CCT/NF‐YB/NF‐YC protein complexes, as predicted by modelling. Jitter plots of the iPTM + PTM scores from CCT/NF‐Y complexes, of four CCT domain proteins related to flowering and coloured by the NF‐YC within the complex. The grey line shows a threshold of iPTM + PTM = 1. The x axis is non functional, provided only for horizontal dispersion of data points.

We tested the stability of macrocomplexes formed between barley CCT domain containing proteins HvVRN2a, HvCO1, HvCO2 and HvPRR37 (PPD1), together with NF‐Y subunits (NF‐YB and NF‐YC) proteins, to bind to the DNA motif CCACA. Overall, the complexes were more stable with HvVRN2a than with HvCO2 or HvCO1 (Figs 6, S7A). The modelled barley CCT proteins bound fittingly to the complex containing NF‐YB and NF‐YC, as confirmed in rice (Fig. S7B). The maximum scores (iPTM + PTM) for each of these three CCT proteins with an NF‐YB/NF‐YC complex were 1.51, 1.37 and 1.37, respectively. Protein PPD1 is predicted to bind to the NF‐Y complex, yet with lower stability (maximum score 1.3). The protein sequence of HvVRN2a was used in the complexes since its orthologue in wheat, ZCCT1, was used by Li *et al*. (2011) in the yeast two‐ and three‐hybrid assays. Similar results were obtained for HvVRN2b with the top 10 combinations of HvVRN2a/NF‐YB/NF‐YC (Table S11). Equivalent protein models tested for wheat produced similar results (Fig. S7C).

Predictions for dimers of all CCT domain proteins, either homo or heterodimers, were also tested. In all cases, they presented low stability.

### *HvVRN2* effect on tillering

Different results pointed at a relationship of the orthologues of the gene *OsPROG1* with *HvVRN2*. They were identified as DEGs by vernalization, grouped in the vernalization correlated modules M18 and M27 and were regulated by the *HvVRN2* genes in the GRN (Tables S3, S10). *PROG1* was associated with tillering in rice (Jin *et al*., 2008; Tan *et al*., 2008). Previous evidence in barley indicated a possible role for *HvVRN2* in tillering patterns (Karsai *et al*., 2006). We investigated this relation using unreported tillering data from an independent experiment (Ochagavía *et al*., 2022). Both NILs, among others, were tested under fully vernalized (60 d) and unvernalized conditions, in similar conditions to the vernalization treatments of 0 and 60 d of the RNAseq experiment. Tiller production showed a linear progression through time, with a linear trend explaining a large proportion of its variance; 84% and 94% for unvernalized and vernalized plants, respectively (Fig. S8; Table S12). However, the pace of tiller production was different for the two genotypes. Fitting separate regression lines per genotype produced significantly different slopes for C01 and C03, for both vernalized and unvernalized plants. C01 produced more tillers than C03, independently of the vernalization treatment (Fig. S8A,B). C03 main shoot apices were more developed than C01 throughout the experiment, indicating faster development (Fig. S8E,F). Interestingly, leaf production in the main tiller progressed in the two genotypes at the same pace in fully vernalized C01 and C03 plants, and for unvernalized C03 (Fig. S8C,D). Therefore, the differences in tiller formation appeared to be decoupled from leaf formation. This was particularly visible for the period up to Z31 in vernalized plants, in which C01 produced more than 2 tillers while C03 produced none, while both presented the same number of leaves in the main stem. These trends resulted in different numbers of tillers per genotype at Z49 stage in the fully vernalized treatment (6.25 and 3.00 for C01 and C03, respectively). At the unvernalized treatment, C03 also produced 3.00 tillers on average at Z49, whereas for C01 unvernalized, tillering continued for a longer period, reaching over 20 tillers in total, as plants did not reach first node stage (Z31) throughout the entire experiment. These results suggest that VRN2 may influence prejointing tillering partly independently of overall leaf developmental progression, although this could be a particular case for the ultrafast C03 in the most inductive conditions.

Linear regressions of leaf production explained 97.9% and 97.7% of the total variance for unvernalized and vernalized conditions, respectively (Fig. S8C,D; Table S12). Tiller production was apparently modulated by vernalization in C03. For the vernalized plants, tiller production did not begin until Z31 (first node detectable) and was sustained even after awn tipping (Z49). For unvernalized plants, however, tillering started before Z31 and was repressed from Z31 until Z49, and resumed thereafter. By contrast, in C01, tillering occurred at a rather constant rate during the experiment. Apparently, the absence of *HvVRN2* affects the period of tillering occurrence, in interaction with vernalization and plant age or growth phase, possibly through the influence on the expression of other genes. It can be concluded that, in this environment and genetic background, *HvVRN2* stabilizes the production of tillers, even in vernalized plants.

## Discussion

### Different patterns of expression between *HvVRN2* genes

This is the first time that different expression levels of the two *HvVRN2* genes, over a wide range of conditions, have been clearly established. Previous reports (Trevaskis *et al*., 2006; Szűcs *et al*., 2007) suggested a predominant role of *HvVRN2b* over *HvVRN2a* as the main repressor in the vernalization pathway. In wheat, although both genes appear to be involved in vernalization (Distelfeld *et al*., 2009), *ZCCT2* (ortholog of *HvVRN2b*) has a larger role (Chen & Dubcovsky, 2012). Our result is consistent with this hypothesis, as both genes produce essentially the same protein, but the transcripts of *HvVRN2b* are clearly more abundant.

According to the standing hypothesis, formulated in wheat (Chen & Dubcovsky, 2012), *HvVRN1* is the repressor of *HvVRN2*. This hypothesis explains well the complete repression of *HvVRN2a* when *HvVRN1* rises. However, it is not sufficient to explain the behaviour of *HvVRN2b*. Even after 60 d of vernalization, and with *HvVRN1* apparently fully expressed, some expression of *HvVRN2b* was still present. Moreover, *HvVRN2b* showed some expression at 60 d of vernalization after 25 d in the growth chamber, while it was fully repressed at 5 and 15 d, hinting at a possible de‐repression (Fig. 3a). Furthermore, the GRN analysis points at possible TFs involved in differential expression of the two *HvVRN2* genes. We cannot conclude that one *HvVRN2* gene is more relevant than the other, but the differences in expression levels and patterns encourage further investigation for potential differences in function and environmental control. The factors responsible for *HvVRN2* induction by partial vernalization (Fig. S5) can be speculated about. We found an induction of *HvPRR37* (*PPD‐H1*) due to vernalization, and the GRN also detected a relationship between *HvPRR37* and both *HvVRN2* genes. Taken together, these facts indicate that *HvPRR37* could be responsible for the induction of *HvVRN2* expression under partial vernalization. This view is consistent with the findings of Mulki & von Korff (2016), who reported a link between the expression of these genes ‘before vernalization’.

Cha *et al*. (2022) also found strikingly different expressions of the *ZCCT1* and *ZCCT2* genes in wheat, leading them to state that ‘…considering the *VRN2* locus as a single gene is potentially misleading in terms of understanding the vernalization response…’. Our findings are consistent with this view. The low but sustained expression of *HvVRN2b* is consistent with other reports of expression of *HvVRN2* under noninductive conditions (Monteagudo *et al*., 2019; Fernández‐Calleja *et al*., 2022). The protracted expression of *HvVRN2b* concurrent with *HvVRN1* and *HvFT1* full expression suggests possible roles of *HvVRN2* loci beyond flowering repression.

### The computed gene regulatory network reflects known interactions and identifies new determinants of flowering

The inclusion of flowering‐related genes provides a positive control for the relationships detected in our experiment. We found a close relationship of *HvVRN1* expression with *HvVRN2a* and *HvVRN2b*, *HvBM3* and *HvBM8* (paralogs of *HvVRN1*), *HvFT1*, *HvFT2* and *HvPRR37* (*PPD‐H1*). Also, both *HvVRN2* genes were connected with *HvPRR37*. All these connections were already described in the literature (Mulki & von Korff, 2016; Shaw *et al*., 2020). Both *HvVRN2* genes were regulated by the other, suggesting a regulatory loop, as already proposed in wheat (Hirsz *et al*., 2026). Their detection in our experiment validates the procedures of analysis and provides a certain confidence over the rest of the findings highlighted in Fig. 5. New connections have also been found in this study, as is the case of a GATA TF or a B3 domain‐containing protein. Some relationships between genes, whose expression and function occur mostly in the apex, were detected in the GRN, which could be an effect of the dominant allele at *PPD‐H1* (Digel *et al*., 2015) or the genetic background. Other known relationships were not detected. For instance, *HvVRN2* and *HvCO2* expression were not directly linked in our study, which is consistent with the hypothesis that they compete at the protein level to bind NF‐Y proteins (Li *et al*., 2011). Moreover, *CONSTANS* expression in *Arabidopsis* is not directly linked with protein quantity. CO protein is stable in late hours under LD photoperiod, as it is degraded during morning and night (Brambilla & Fornara, 2017).

As mentioned, *HvSNF2* and *HvVRN2a* are adjacent on the long arm of chromosome 4H. Gene *HvSNF2* has a single copy in barley (Yan *et al*., 2002). This gene seems to perform essential tasks related to chromatin remodelling during cell division. A regulatory role in barley spike development has been suggested (Chen *et al*., 2023), although its expression has been reported in root, spike and leaves. In this last case, higher expression was linked to cold treatments, consistent with our study. Interestingly, *HvSNF2* is only expressed in CSIRO01, implying the necessity of the presence of *HvVRN2* to be expressed, at least in leaves in these conditions. *HvSNF2* and *HvVRN2a* showed divergent orientations in the accessions of the pangenome (Fig. S6). This structure could result in simultaneous expression either by the action of a common promoter region between the starting points of both genes, being regulated by the same factors as *HvVRN2*, or by the opening of chromatin for the entire region. Allelic differences were detected between both genotypes, which could also affect their expression.

*HvVRN1* plays a central role in the GRN, interacting with the largest number of genes. Our transcriptome results support previous findings of genes controlled by VRN1, based on chromatin immunoprecipitation (Deng *et al*., 2015). Besides *HvFT1* or *HvVRN2*, genes in co‐expression modules M18 and M27 (a *bHLH* TF, both *OsPROG1* orthologs, *CBF*s, an *AP2/B3* TF, the MADS‐box *HvVRT2* and the identified novel bZIP *HvFD1‐like* gene) were in common with that study.

There are differences among the genes located one degree of separation from *HvVRN2a* and *HvVRN2b*, suggesting different pathways of regulation. While *HvVRN2a* seems closer to *HvFT1* and *HvVRN1*, *HvVRN2b* showed more connections to TF not related to the flowering and vernalization pathway. For example, it seems to regulate two TF within the module M3, enriched with GO terms related to chloroplasts and photosystems. *HvVRN2b* could have a function beyond the flowering and vernalization pathway, as its expression continues even after the florigen signal of *HvFT1* is activated.

### Vernalization‐related co‐expression modules identify new candidate genes for flowering and tiller production

The high and opposite correlation coefficients between modules M18 and M20 with vernalization suggested an antagonistic relationship, pointing to the function and time of expression of the modules. M18 most likely clusters genes expressed before *HvFT1* as a response to hormones. Genes within M29, such as *HvVRN2b*, or nonclustered genes, such as *HvVRN2a*, work with M18 genes and do not allow the vegetative to reproductive transition. When there is enough vernalization, M20 genes are activated, repressing M18 and M29 function, allowing the transition to the reproductive phase.

Our research has unravelled new genes with possible function in the barley vernalization pathway. One of them is an ortholog of *Oryza sativa*'s BZIP77 (*OsFD1*). It is present in M18, clearly affected by the application of vernalization. FD protein forms a complex with FT1 and 14‐3‐3 proteins that transduces the flowering signal from the leaves to the shoot apex, as shown in *Arabidopsis* (Abe *et al*., 2005; Wigge *et al*., 2005) and rice (Taoka *et al*., 2011). We have detected expression of this gene related to those of *HvVRN1*, *HvVRN2a* and *HvFT1*, indicating a close relationship with the vernalization response. The *FD‐like* gene detected in this study was not included in the *FD‐like* genes reported by Li *et al*. (2015), whose proteins formed complexes with FT and 14–3‐3. Another gene newly identified, the TF AP2/B3, is an orthologue of flowering repressors from the RAV gene family described in *Arabidopsis* (Hu *et al*., 2021) and rice (Osnato *et al*., 2020).

Another interesting gene identified in this study is a zinc‐finger protein, orthologue of rice *PROG1*, a gene related to leaf angle and tiller number predominantly expressed in axillary meristems (Jin *et al*., 2008; Tan *et al*., 2008). *OsPROG1* shows several orthologous genes in barley, all located in tandem repeats. Two *HvPROG1* genes were detected as DEGs and were clustered by co‐expression in modules M18 and M27, both negatively correlated with vernalization time. *HvPROG1‐2*, in particular, presents expression patterns compatible with the differences observed for tiller production between genotypes and treatments. *HvPROG1* genes are annotated as zinc‐finger TF but our analyses did not classify them as TF, and were not considered as regulators in the GRN analysis. Still, the two *HvPROG1* are regulated both by *HvVRN1* and by *HvVRN2*, as well as other TFs shown in Fig. 5. Karsai *et al*. (2006), in a facultative × winter barley population, showed that tiller production is higher in presence of *HvVRN2*. Furthermore, Hirsz *et al*. (2026), in *VRN2* tilling mutants of *T. aestivum*, identified an effect of *VRN2‐D2* on early tiller development. An effect of the rice orthologue of *HvVRN2*, *Ghd7*, on tillering has also been reported (Weng *et al*., 2014; Guo *et al*., 2025). In this research, we have also identified differences in tiller production per day, and tiller number at Z31 and Z49 (Fig. S8). Altogether, *HvVRN2* could be an enhancer of the *HvPROG1* genes in the basal section of the leaf, promoting tillering.

### Higher stability of VRN2/NF‐Y complexes as limiting CO2/NF‐Y activation of the flowering process

Brambilla & Fornara (2017), discussing the combinatorial properties of the NF‐Y complex, hypothesized that it could interact not only with NF‐YA or CO‐like proteins but also with other CCT domain proteins in general. This was proven for rice GHD7‐CCT, GHD8 (also named OsNF‐YB11) and OsNFYC7 (Shen *et al*., 2020).

These observations are compatible with the competition between CO2 and VRN2 described in wheat (Li *et al*., 2011; Shaw *et al*., 2020), and the possible differences in function of each complex. All these trimers can bind DNA through the CCT domain and also form larger complexes among themselves, binding through their zinc‐fingers. The structural analysis indicates that the zinc‐finger is not the likely point of union to DNA. Rather, it is more likely that the CCT domain is the DNA binding domain, recognizing the CCACA motif present in promoter regions (Shen *et al*., 2020).

Structural modelling suggests that the VRN2/NF‐Y complex may be more stable than the CO2/NF‐Y. Whether this is indeed the case *in planta*, and how the potential co‐existence of both complexes may influence their interaction dynamics with promoter regions of target genes such as *HvFT1*, remains to be elucidated. It is nevertheless interesting to note that, in the model plant *A. thaliana*, the two B‐box (zinc finger) domains of CO form a continuous head‐to‐tail oligomer‐like structure, further stabilized by the binding of the CO/NF‐Y complexes through the CCT domains to the *HvFT1* promoter, inducing its expression (Zeng *et al*., 2022; Huang *et al*., 2025). In barley, the stability of this complex structure could be affected by the lesser number of zinc‐finger domains carried by VRN2 (only one) and PPD1 (none), not allowing the head‐to‐tail binding structure, hence affecting *HvFT1* expression. VRN2 could compete with CO2 to bind NF‐Y and, additionally, the presence of VRN2/NF‐Y concurrently with CO2/NF‐Y could weaken the stability of the protein complexes binding to the *HvFT1* promoter. Moreover, the differences in transcript quantity between *HvVRN2* genes could be explained due to an extra CCACA DNA motif present in the promoter of *HvVRN2b*, compared with *HvVRN2a* (Fig. S9). This extra binding site could allow a more stable oligomerization of the CCT/NF‐Y complex, which could explain the differences in expression levels and dynamics between the *HvVRN2* genes (Fig. 3a).

Summarizing, differences in expression of both *HvVRN2* genes have been identified. *HvVRN2a* gene expression is lower than that of *HvVRN2b*, as recently confirmed by Zhu & Stein (2025). While both *HvVRN2* genes seem to regulate the flowering pathway, a GRN identified connections outside the florigen signal of *HvFT1*, mostly for *HvVRN2b*. While both *HvVRN2* have a redundant repressor effect in flowering, they should be considered different genes. The expression of the *HvVRN2* locus increases during the accumulation of days under LD photoperiod, after insufficient vernalization, suggesting a possible interaction with *HvPRR37 (PPD‐H1)*. While CCT proteins (including VRN2) compete for the NF‐Y complex, VRN2/NF‐Y complexes were predicted to be more stable. The higher stability of VRN2/NF‐Y complexes could block the CO2/NF‐Y oligomerization required to induce *HvFT1*. Moreover, the higher tillering of winter barley could be related to *HvPROG1* genes, induced by *HvVRN2*.

The induction of *HvVRN2* expression with partial vernalization may have an agronomic effect in field conditions. The occurrence of this effect will depend on the balance of the environmental stimuli affecting these genes. If vernalization is not complete before daylength increase induces *HvVRN2* expression, further effects on both development and plant architecture may occur. In fact, *HvVRN2* has recently been found in a large number of spring varieties for no apparent reason (Montardit‐Tarda *et al*., 2025; Zhu & Stein, 2025). The effect on tillering found in our study could underlie this unexplained retention of *HvVRN2* in many spring varieties.

In conclusion, the *ZCCT* genes inside locus *HvVRN2* should be considered as different genes and could have different potential roles during vernalization. The repression of flowering by locus *HvVRN2* may take place at the protein level, more likely preventing the required oligomerization of CO2/NF‐YB/NF‐YC protein complexes at the promoter region of *HvFT1*. *HvVRN2* has a potential role in tiller production, which could translate into higher yields. Further research on *HvVRN2* should consider the differences between the *ZCCT* genes.

## Competing interests

None declared.

## Author contributions

Funding was acquired by AMC, BC‐M, EI, FM‐T and IK. The experiment was designed by AMC, EI, FM‐T and IK. Plant growth and wet‐lab were performed by AMC, FM‐T and IK. Dry‐lab methodology was designed by BC‐M, FM‐T and PB. Data was analysed by FM‐T and IP. Research was supervised by AMC, BC‐M, EI, IK and PB. Summarized data and graphics were created by AMC, BC‐M, FM‐T, IP and EI. The original draft was prepared by AMC, FM‐T and EI. All authors revised and approved the final manuscript.

## Disclaimer

The New Phytologist Foundation remains neutral with regard to jurisdictional claims in maps and in any institutional affiliations.

## Supporting information


**Dataset S1.** Protein models computed by Alphafold.

**Fig. S1.** Photos of unvernalized plants, showing differences in development.
**Fig. S2** Map of genetic introgressions between C01 and C03.
**Fig. S3** Regression analyses of *HvVRN* genes' expression over time.
**Fig. S4** Gene expressions of TF presented in the GRN in Fig. 5.
**Fig. S5** Diagram of temporal relationship of gene expression between selected vernalization modules and genes.
**Fig. S6** Pangenome occupancy of *HvSNF2* and *HvVRN2*.
**Fig. S7** Scatter plot of barley and wheat CCT/NF‐Y modelled protein complexes and 3D structure of VRN2a/NF‐Y complex.
**Fig. S8** Regression analyses of main shoot's tillers and leaves, and apex development, over time.
**Fig. S9** Location of CCACA motif in the promoters of CCT genes.

**Table S1** Sample information and sequencing data.
**Table S2** Genotyping of both NILs (50 K and RNASeq variant calling).
**Table S3** Expression data (tpm) at gene‐level for all samples, differentially expressed genes results, and co‐expression module membership.
**Table S4** Flowering‐related gene names and gene model ID in BaRT2v18 transcriptome.
**Table S5** Protein sequences and oligonucleotide with CCACA DNA‐motif for modelling prediction.
**Table S6** Regression analyses of *HvVRN* genes’ expression over time.
**Table S7** Gene Ontology enrichment for each co‐expression module.
**Table S8** Identified transcription factors in the reference transcriptome.

**Table S9** Gene Regulatory Network analysis.
**Table S10** Summary of vernalization‐related TF Regulatory Network.
**Table S11** Modelling scores of CCT/CCT dimers and CCT/NF‐Y protein complexes.
**Table S12** Regression analyses of tillers and leaves in the main shoot over time.
Please note: Wiley is not responsible for the content or functionality of any Supporting Information supplied by the authors. Any queries (other than missing material) should be directed to the *New Phytologist* Central Office.

## Data Availability

All sequencing reads are available under study no. PRJEB85891 in the European Nucleotide Archive. Scripts are available at https://github.com/chesQhub/barley_vernalisation_rnaseq. All predicted protein models are available at Dataset S1.
