# Supplementary material for: Vernalization regulatory network identifies potential novel functions for genes in the HvVRN2 locus
Source: New Phytol. 2026 Apr 12;250(6):4036–53. doi: 10.1111/nph.71162 (PMC13193379; doi:10.1111/nph.71162)
Supplement: Supplementary file 2 — Fig. S1. Photos of unvernalized plants, showing differences in development. Fig. S2 Map of genetic introgressions between C01 and C03. Fig. S3 Regression analyses of HvVRN genes' expression over time. Fig. S4 Gene expressions of TF presented in the GRN in Fig. 5. Fig. S5 Diagram of temporal relationship of gene expression between selected vernalization modules and genes. Fig. S6 Pangenome occupancy of HvSNF2 and HvVRN2. Fig. S7 Scatter plot of barley and wheat CCT/NF‐Y modelled protein complexes and 3D structure of VRN2a/NF‐Y complex. Fig. S8 Regression analyses of main shoot's tillers and leaves, and apex development, over time. Fig. S9 Location of CCACA motif in the promoters of CCT genes. [file NPH-250-4036-s001.docx]

## *New Phytologist* Supporting Information

Article title: Vernalization regulatory network identifies potential novel functions for genes in the *HvVRN2* locus

Authors: Francesc Montardit-Tarda, Irene Puyó, Bruno Contreras-Moreira, Ildikó Karsai, Philippa Borrill, Ana M Casas, Ernesto Igartua

Article acceptance date: 11 March 2026.

The following Supporting Information is available for this article:

**Fig. S1.** Photos of unvernalized plants, showing differences in development

**Fig. S2.** Map of genetic introgressions between C01 and C03

**Fig. S3.** Regression analyses of *HvVRN* genes’ expression over time

**Fig. S4.** Gene expressions of TF presented in the GRN in Figure 5

**Fig. S5.** Diagram of temporal relationship of gene expression between selected vernalization modules and genes

**Fig. S6.** Pangenome occupancy of *HvSNF2* and *HvVRN2*

**Fig. S7.** Scatter plot of barley and wheat CCT/NF-Y modelled protein complexes and 3D structure of VRN2a/NF-Y complex

**Fig. S8.** Regression analyses of main shoot’s tillers and leaves, and apex development, over time

**Fig. S9.** Location of CCACA motif in the promoters of CCT genes

**Table S1.** Sample information and sequencing data

**Table S2.** Genotyping of both NILs (50K and RNASeq variant calling)

**Table S3.** Expression data (tpm) at gene-level for all samples, differentially expressed genes results, and co-expression module membership

**Table S4.** Flowering-related gene names and gene model ID in BaRT2v18 transcriptome

**Table S5.** Protein sequences and oligonucleotide with CCACA DNA-motif for modelling prediction

**Table S6.** Regression analyses of *HvVRN* genes’ expression over time

**Table S7.** Gene Ontology enrichment for each co-expression module

**Table S8.** Identified transcription factors in the reference transcriptome

**Table S9.** Gene Regulatory Network analysis

**Table S10.** Summary of vernalization-related TF Regulatory Network

**Table S11.** Modelling scores of CCT/CCT dimers and CCT/NF-Y protein complexes

**Table S12.** Regression analyses of tillers and leaves in the main shoot over time

**Dataset S1.** Protein models computed by Alphafold

**Supporting Figures**

**Fig. S1** Unvernalized plants grown under long photoperiod (16/8 h light/dark) for 25 days. A) CSIRO01 plants, with *HvVRN2*, which did not reach jointing stage (Z31). B) CSIRO03 plants, without *HvVRN2*, which are already extending flag leaf (Z41). Photos of dissected apices are placed at the right of each genotype panel, showing apices at 0 days (top) and at 13 days (bottom) after growing under long photoperiod.


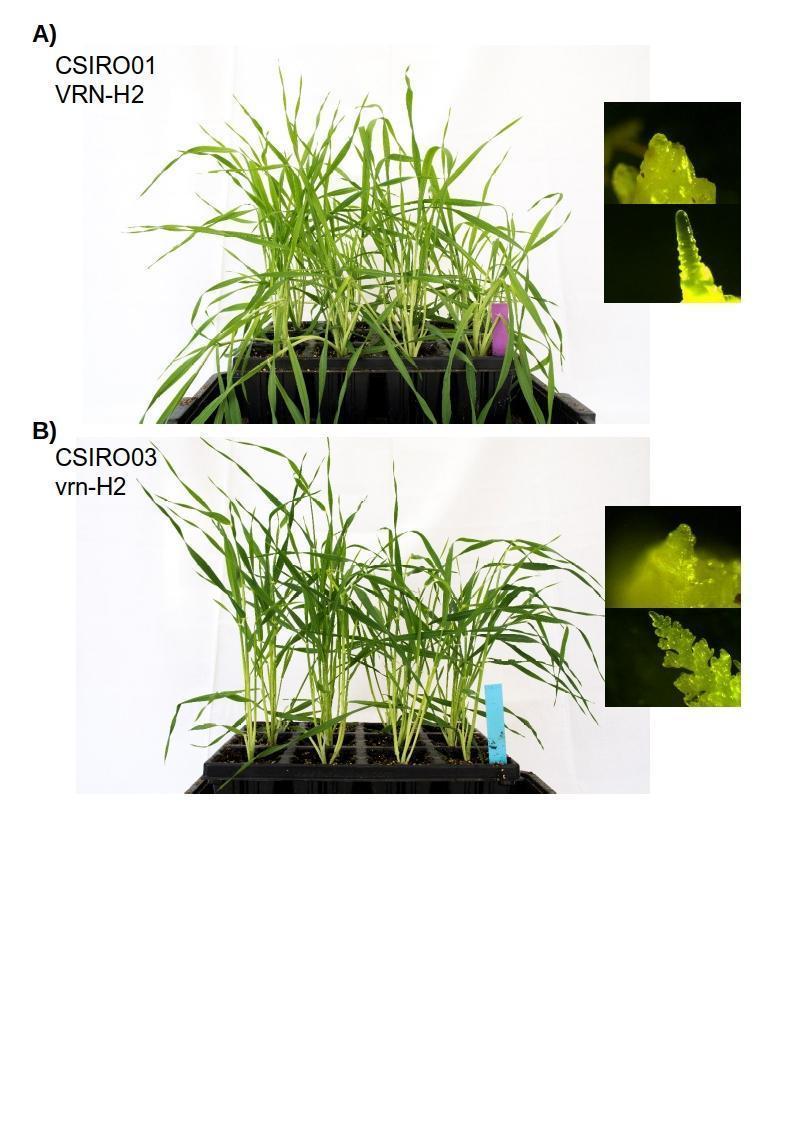


**Fig. S2.** Introgressed regions in CSIRO01 near-isogenic line, compared to CSIRO03. Chromosomic regions with at least one SNP are colored with green. Blue bars indicate the number of SNPs in each region.


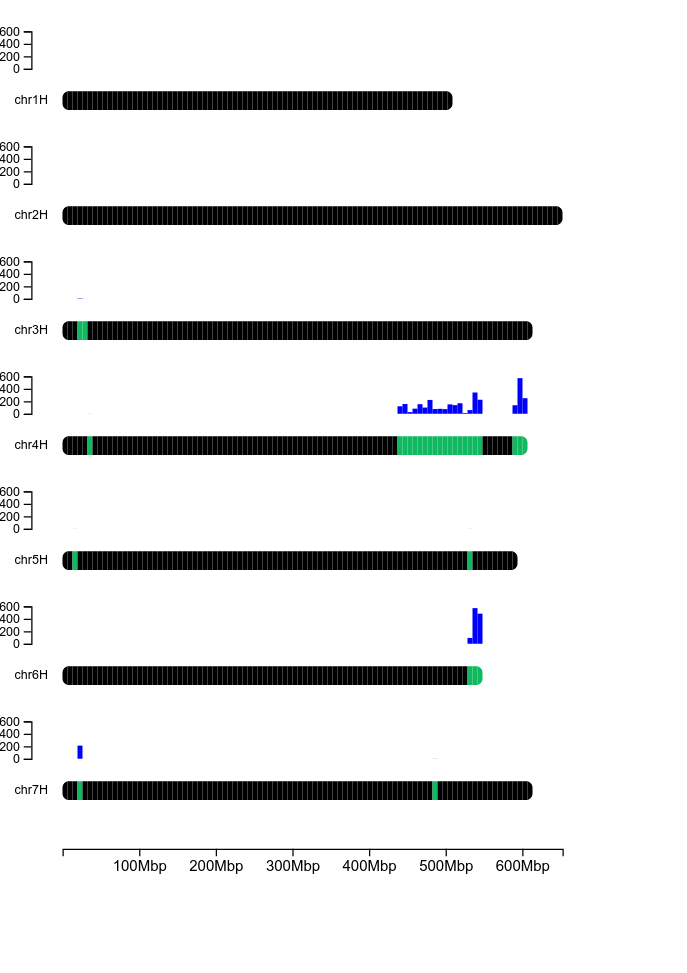


**Fig. S3.** Regression analyses of *HvVRN* genes’ expression (in tpm) in C01 genotype over growing days. A) *HvVRN2a*, B) *HvVRN2b*, C) *HvVRN1*, colored by vernalization time and each dot corresponding to a sample. Regression analysis results can be found in Table S6, below (p. 13).


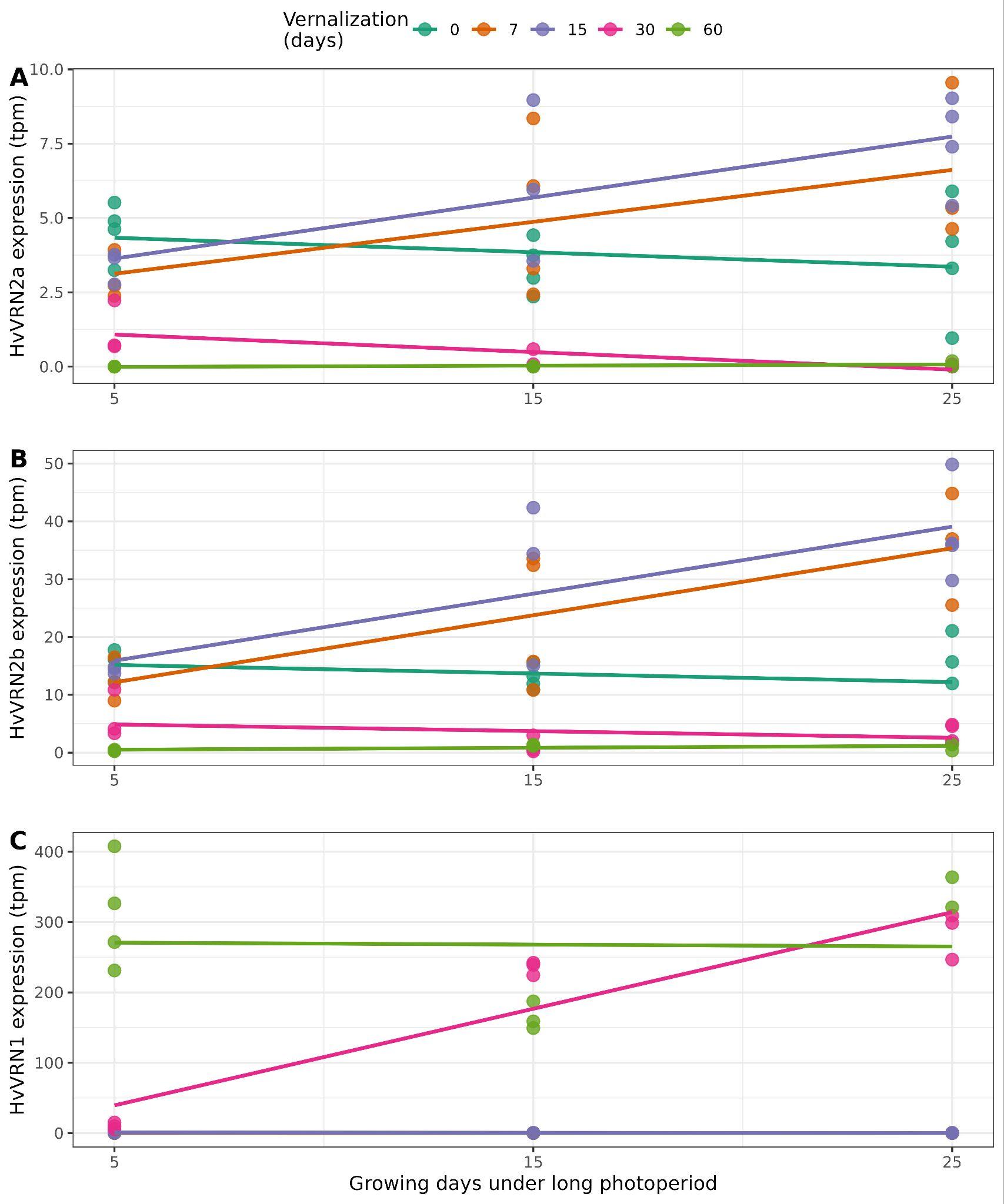


**Fig. S4.** Gene expression of TF presented in the vernalization GRN. Panels divide genes by module, corresponding to A) M0, B) M29, C) M20, D) M18, E) M4, F) M27, G) M6. Horizontal panels divide NILs and vertical panels each vernalization time. *HvVRN1*, *HvVRN2a*, *HvVRN2b* are displayed in all panels as reference.


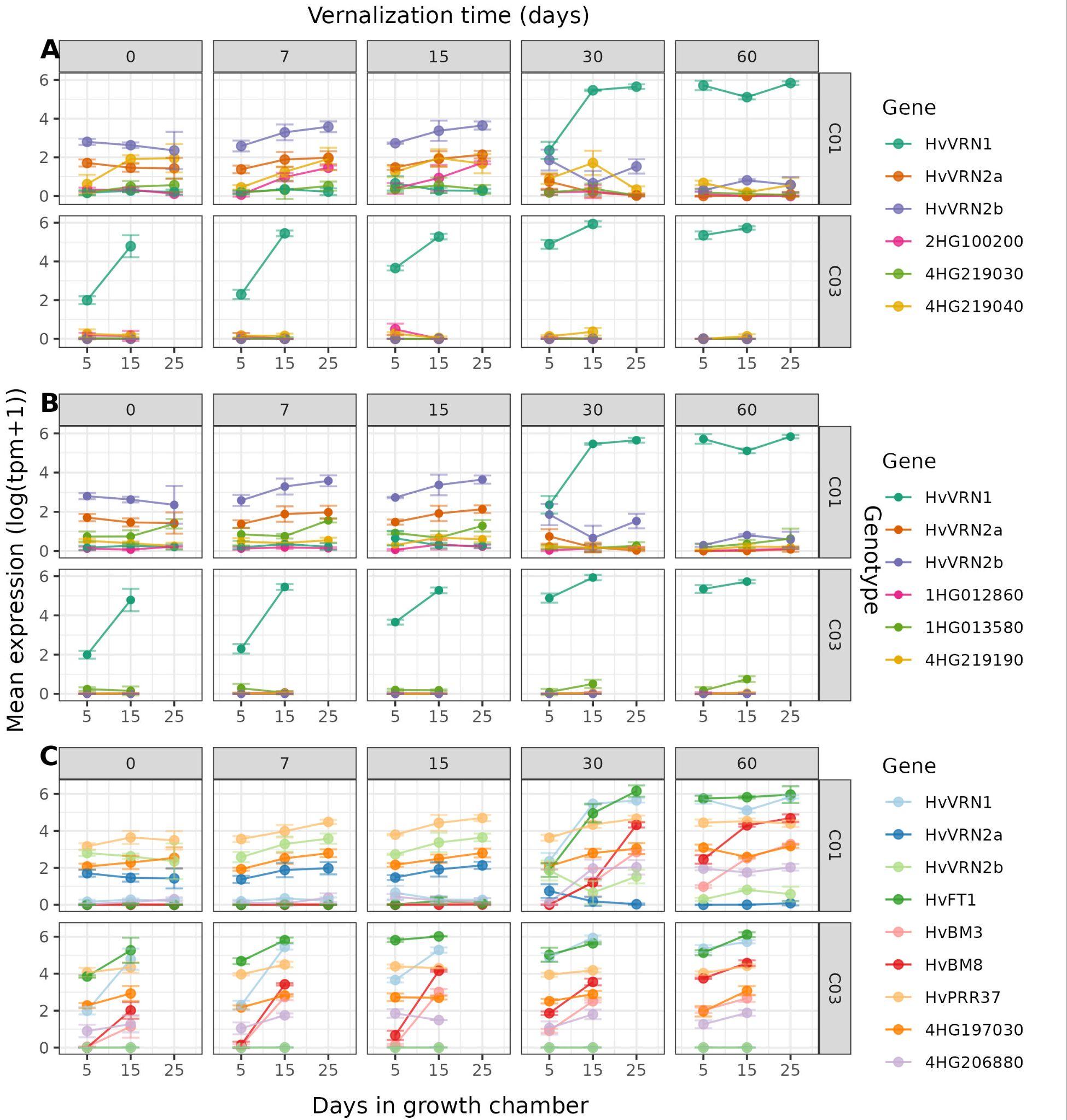


**Fig. S4.** Continued.


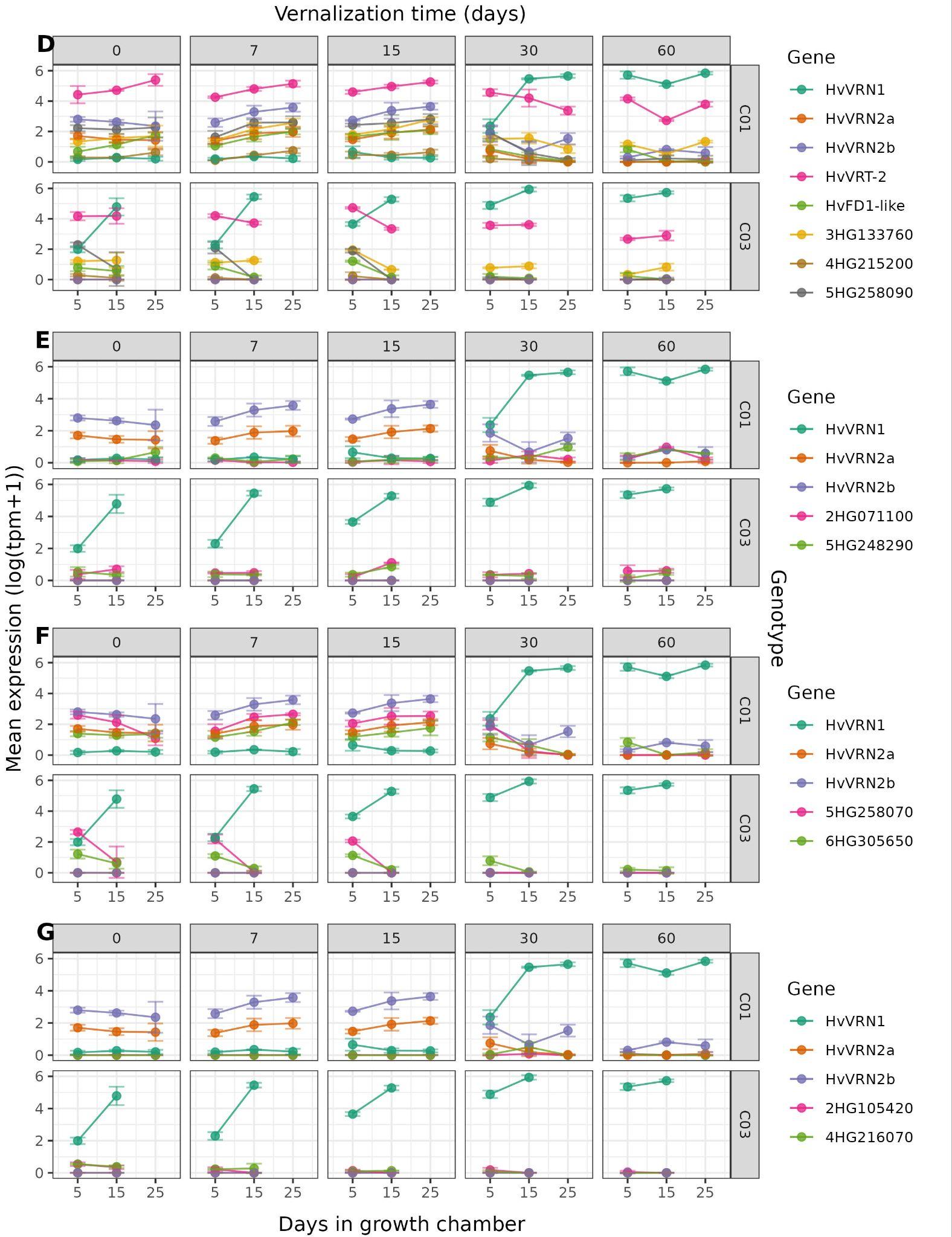


**Fig. S5.** Diagram of temporal relationships of gene expression between selected vernalization modules and genes. Arrows (blue or white) indicate induction whereas red blunt lines indicate repression. Modules M12, M19 and 27 are clustered together by their correlations, whereas M18 is independent.


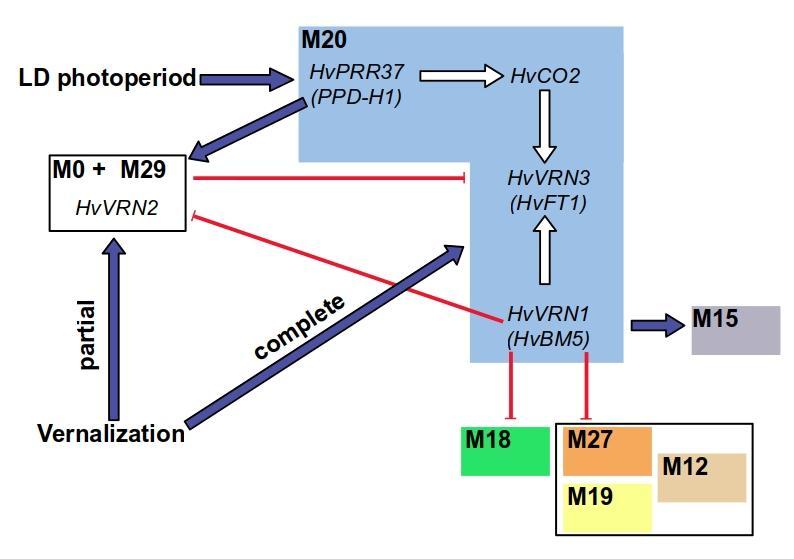


**Fig. S6.** Occupancy of *HvSNF2* (green) and HvVRN2a (magenta) in the barley pangenome v1. The gene immediately at the right of *HvSNF2* is *HvVRN2a*, located in the complementary DNA strand. The region is inverted in some accessions, compared to MorexV3. *HvSNF2* and *HvVRN2a* are always present on opposite strands. Figure produced with software GET_PANGENES (https://github.com/Ensembl/plant-scripts) and pyGenomeViz (https://pypi.org/project/pygenomeviz)


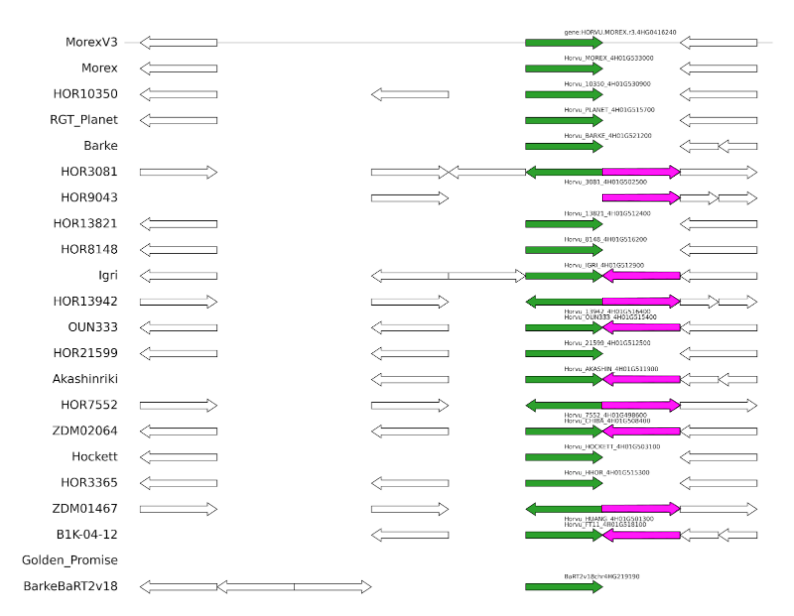


**Fig. S7.** Protein modelling of CCT/NF-YB/NF-YC complexes. A) Scatter plot of iPTM + PTM scores from barley CCT/NF-Y complexes, coloured by the NF-YB protein in the complex. The red line shows a threshold of iPTM + PTM = 1. B) Structure of the best VRN2/NF-YB/NF-YC complex, bound to a CCACA DNA-motif through the CCT domain. C) Scatter plot of iPTM + PTM scores from CCT/NF-Y complexes of *Triticum* species, coloured and shaped by NF-Y proteins.


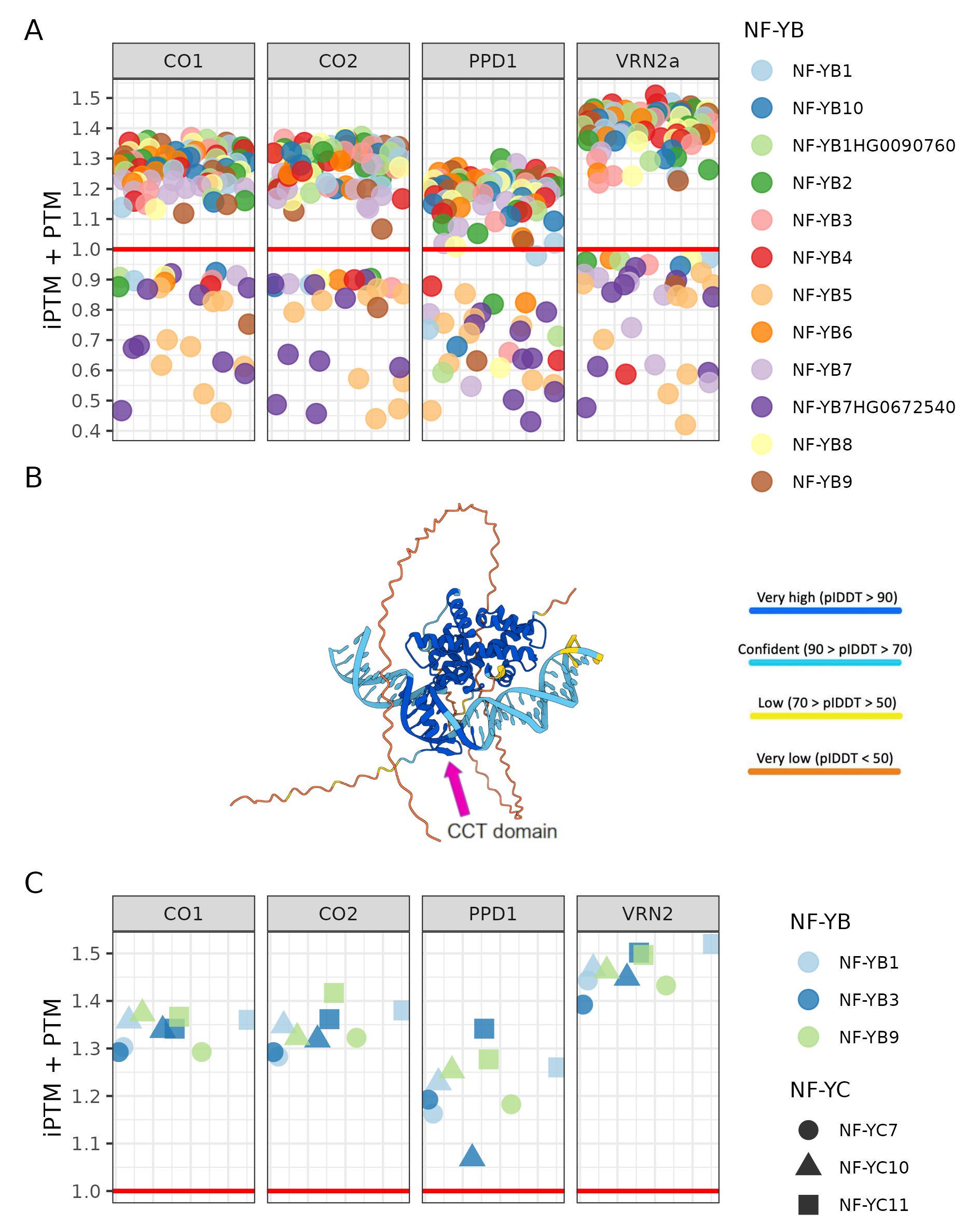


**Fig. S8.**
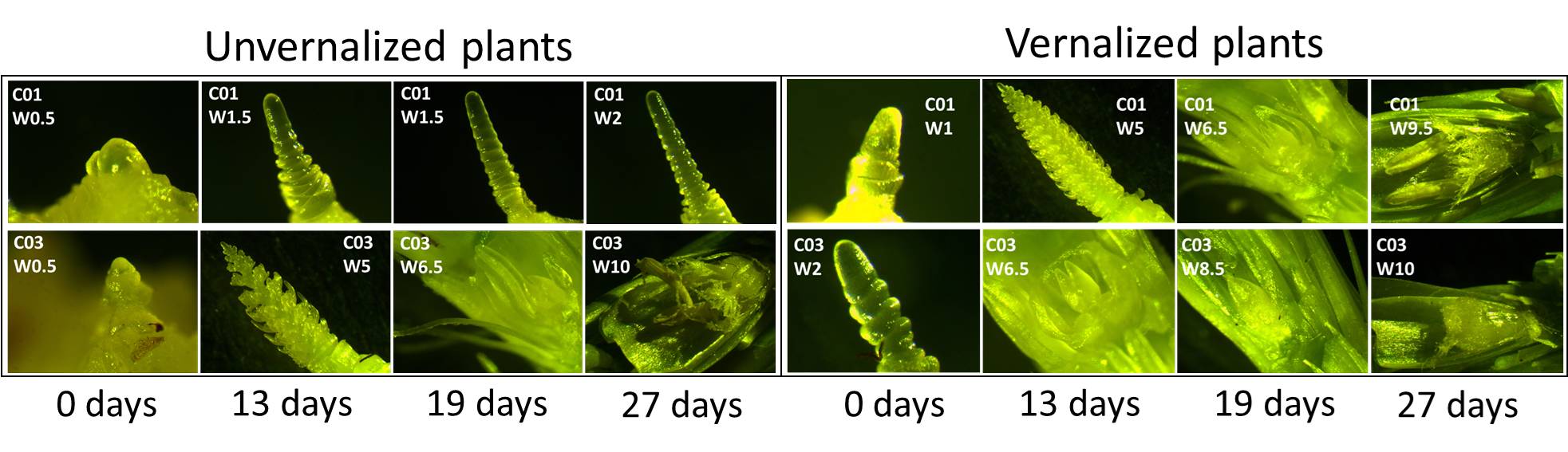
 Regression analyses of main shoot’s tillers and leaves, and apex development, over time. C01 is coloured in red and C03 in blue. Vertical lines indicate Z31 (solid) or Z49 (dashed). Tiller production by day in A) unvernalized and B) vernalized of 4 plants, and leaf number on the main tiller in C) unvernalized and D) vernalized of the same 4 plants. Main shoot apices, indicating Waddington stage, throughout days for E) unvernalized and F) vernalized plants.


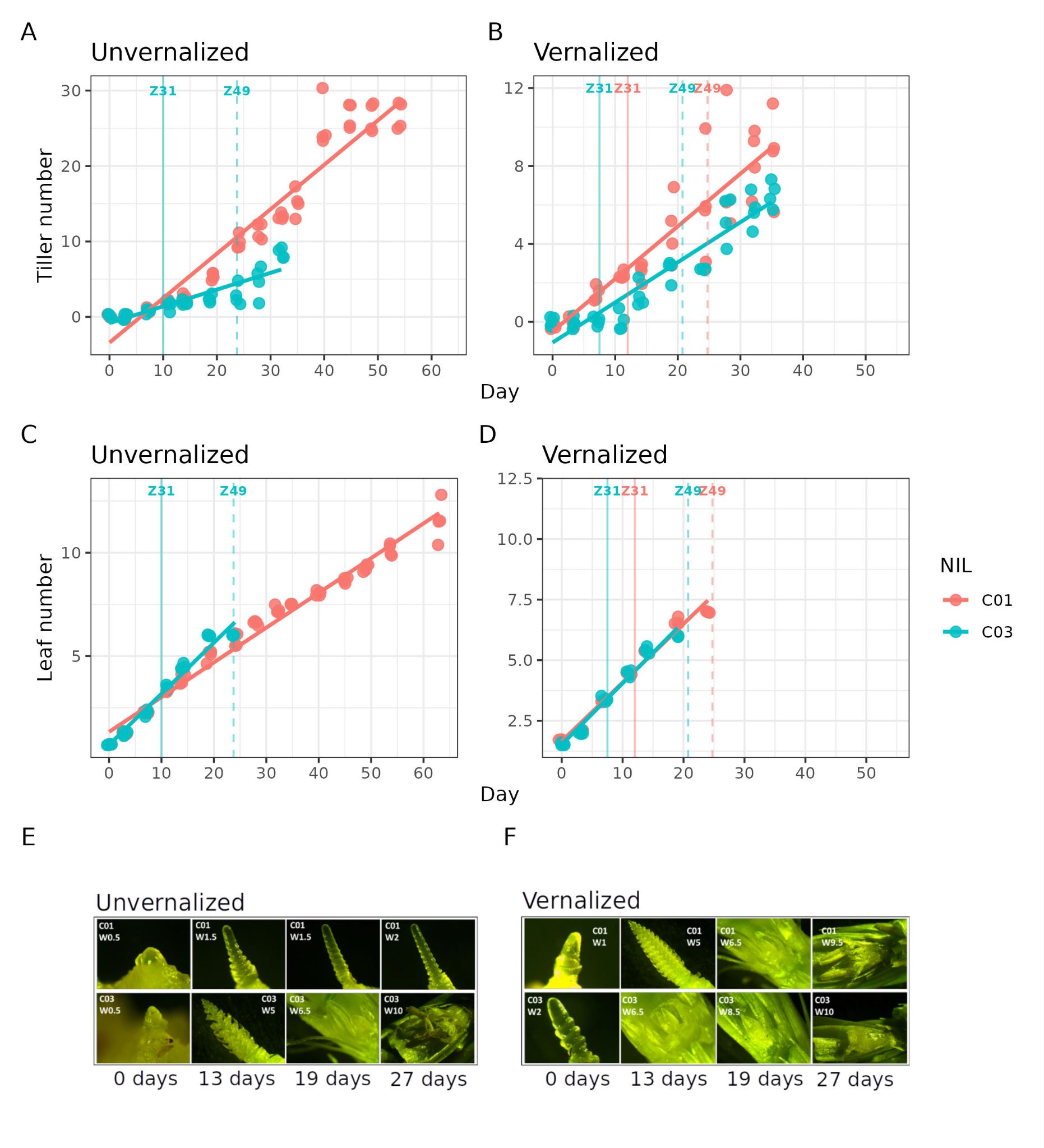
**Fig. S9.** Position of CCACA motif in the promoter regions (-1000b bp, 0 bp) from the start of several genes encoding CCT domain proteins and FT1. Sites on the reverse strand are shown below the promoter lines, extracted from the genome of HOR_13942 of the pangenome v1.


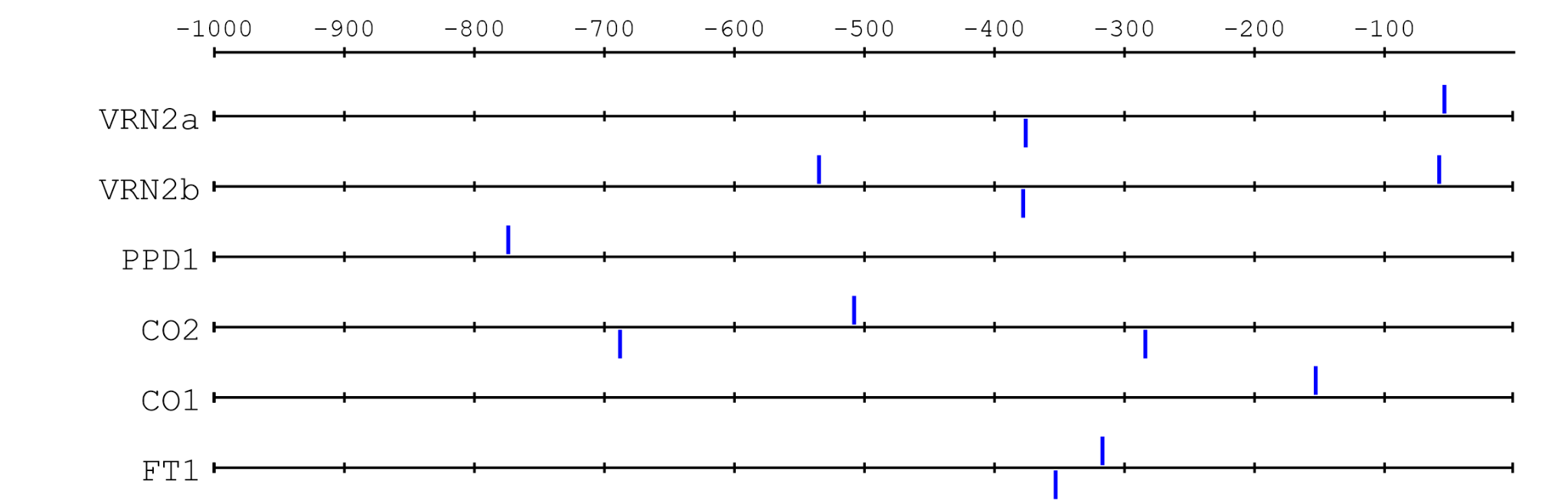


**Table S6**. Results of regression analyses of *HvVRN1*, *HvVRN2a* and *HvVRN2b* gene expression over time. A separate regression line per vernalization treatment was fitted, only for C01. Each intercept and slope was compared to the non-vernalization line (0 days of vernalization), considered as reference.

| **Parameter** | **estimate** | **s.e.** | **t** | **t pr.** |
| --- | --- | --- | --- | --- |
|  | *HvVRN1* | | | |
| Intercept V0 | 0.2 | 28.1 | 0.01 | 0.994 |
| Slope V0 | 0.0 | 1.6 | 0.00 | 0.999 |
| Difference intercept V7-V0 | 0.0 | 42.6 | 0.00 | 1.000 |
| Difference intercept V15-V0 | 0.8 | 42.5 | 0.02 | 0.984 |
| Difference intercept V30-V0 | -29.4 | 42.9 | -0.69 | 0.497 |
| Difference intercept V60-V0 | 271.9 | 40.8 | 6.66 | <0.001 |
| Difference slope V7-V0 | 0.0 | 2.5 | 0.00 | 1.000 |
| Difference slope V15-V0 | 0.0 | 2.4 | -0.01 | 0.988 |
| Difference slope V30-V0 | 13.7 | 2.5 | 5.46 | <0.001 |
| Difference slope V60-V0 | -0.3 | 2.6 | -0.11 | 0.915 |
|  | *HvVRN2a* | | | |
| Intercept V0 | 4.58 | 0.868 | 5.28 | <0.001 |
| Slope V0 | -0.05 | 0.051 | -0.96 | 0.343 |
| Difference intercept V7-V0 | -2.33 | 1.320 | -1.77 | 0.084 |
| Difference intercept V15-V0 | -1.97 | 1.310 | -1.50 | 0.141 |
| Difference intercept V30-V0 | -3.21 | 1.330 | -2.42 | 0.020 |
| Difference intercept V60-V0 | -4.61 | 1.260 | -3.66 | <0.001 |
| Difference slope V7-V0 | 0.22 | 0.078 | 2.88 | 0.006 |
| Difference slope V15-V0 | 0.25 | 0.075 | 3.40 | 0.002 |
| Difference slope V30-V0 | -0.01 | 0.078 | -0.13 | 0.897 |
| Difference slope V60-V0 | 0.05 | 0.079 | 0.67 | 0.509 |
|  | *HvVRN2b* | | | |
| Intercept V0 | 15.92 | 3.800 | 4.19 | <0.001 |
| Slope V0 | -0.15 | 0.222 | -0.67 | 0.506 |
| Difference intercept V7-V0 | -9.55 | 5.760 | -1.66 | 0.105 |
| Difference intercept V15-V0 | -5.81 | 5.750 | -1.01 | 0.318 |
| Difference intercept V30-V0 | -10.47 | 5.800 | -1.80 | 0.079 |
| Difference intercept V60-V0 | -15.58 | 5.520 | -2.82 | 0.007 |
| Difference slope V7-V0 | 1.31 | 0.340 | 3.85 | <0.001 |
| Difference slope V15-V0 | 1.31 | 0.327 | 4.00 | <0.001 |
| Difference slope V30-V0 | 0.03 | 0.340 | 0.10 | 0.922 |
| Difference slope V60-V0 | 0.18 | 0.347 | 0.53 | 0.602 |

**Table S12.** Results of regression analyses of tiller and leaf production (in the main tiller) over time. A separate regression line per genotype was fitted, and their parameters are compared, using genotype C01 as reference for tillers, in vernalized and unvernalized plants, and for leaves, in vernalized and unvernalized plants.

| **Parameter** | **estimate** | **s.e.** | **t** | **t pr.** |
| --- | --- | --- | --- | --- |
|  | TILLERS-vernalized plants | | | |
| Intercept C01 | -0.495 | 0.351 | -1.41 | 0.162 |
| Slope C01 | 0.270 | 0.017 | 16.05 | <0.001 |
| Difference Intercept C01-C03 | -0.569 | 0.496 | -1.15 | 0.255 |
| Difference Slope C01-C03 | -0.064 | 0.024 | -2.69 | 0.009 |
|  | TILLERS-unvernalized plants | | | |
| Intercept C01 | -3.406 | 0.561 | -6.07 | <0.001 |
| Slope C01 | 0.589 | 0.018 | 32.33 | <0.001 |
| Difference Intercept C01-C03 | 2.587 | 0.878 | 2.95 | 0.004 |
| Difference Slope C01-C03 | -0.367 | 0.041 | -9.05 | <0.001 |
|  | LEAVES IN MAIN TILLER-vernalized plants | | | |
| Intercept C01 | 1.659 | 0.092 | 18.10 | <0.001 |
| Slope C01 | 0.242 | 0.007 | 36.10 | <0.001 |
| Difference Intercept C01-C03 | -0.136 | 0.135 | -1.01 | 0.320 |
| Difference Slope C01-C03 | 0.011 | 0.011 | 0.97 | 0.338 |
|  | LEAVES IN MAIN TILLER-unvernalized plants | | | |
| Intercept C01 | 1.333 | 0.107 | 12.50 | <0.001 |
| Slope C01 | 0.168 | 0.003 | 53.40 | <0.001 |
| Difference Intercept C01-C03 | -0.621 | 0.183 | -3.40 | 0.001 |
| Difference Slope C01-C03 | 0.079 | 0.011 | 6.97 | <0.001 |
